# Till death do us part: the effect of marital status on health care utilization and costs at end-of-life. A register study on all colorectal cancer decedents in Norway between 2009 and 2013

**DOI:** 10.1186/s12913-019-4794-6

**Published:** 2020-02-13

**Authors:** Gudrun Maria Waaler Bjørnelv, Bjørn Edwin, Åsmund Avdem Fretland, Partha Deb, Eline Aas

**Affiliations:** 1 The Intervention Centre, Oslo University Hospital, Postboks 4950 Nydalen, 0424 Oslo, Norway; 2 Institute of Health and Society, University of Oslo, Oslo, Norway; 3 Department of Hepato-Pancreato-Biliary Surgery, Oslo University Hospital, Oslo, Norway; 4 Institute of Clinical Medicine, University of Oslo, Oslo, Norway; 5 Department of Economics, Hunter College, CUNY and NBER, New York, USA; 6 Health Service Research Unit, Akershus University Hospital, Lørenskog, Norway

## Abstract

**Background:**

Economic analyses of end-of-life care often focus on single aspects of care in selected cohorts leading to limited knowledge on the total level of care required to patients at their end-of-life. We aim at describing the living situation and full range of health care provided to patients at their end-of-life, including how informal care affects formal health care provision, using the case of colorectal cancer.

**Methods:**

All colorectal cancer decedents between 2009 and 2013 in Norway (*n* = 7695) were linked to six national registers. The registers included information on decedents’ living situation (days at home, in short- or long-term institution or in the hospital), their total health care utilization and costs in the secondary, primary and home- and community-based care setting. The effect of informal care was assessed through marital status (never married, currently married, or previously married) using regression analyses (negative binominal, two-part models and generalized linear models), controlling for age, gender, comorbidities, education, income, time since diagnosis and year of death.

**Results:**

The average patient spent four months at home, while he or she spent 27 days in long-term institutions, 16 days in short-term institutions, and 21 days in the hospital. Of the total costs (~NOK 400,000), 58, 3 and 39% were from secondary carers (hospitals), primary carers (general practitioners and emergency rooms) and home- and community-based carers (home care and nursing homes), respectively. Compared to the never married, married patients spent 30 more days at home and utilized less home- and community-based care, but more health care services at the secondary and primary health care level. Their total healthcare costs were significantly lower (−NOK 65,621) than the never married. We found similar, but weaker, patterns for those who had been married previously.

**Conclusion:**

End-of-life care is primarily provided in the secondary and home-and community-based care level, and informal caregivers have a substantial influence on formal end-of-life care provision. Excluding aspects of care such as home and community-based care or informal care in economic analyses of end-of-life care provides a biased picture of the total resources required, and might lead to inefficient resource allocations.

## Background

The health care costs at end-of-life are substantial, consequently, there is increasing interest in the health care utilization and costs at patients’ end-of-life [1–3]. People generally express a preference towards being cared for (and dying) at home at their end-of-life [4–7]. To fulfil this wish, the focus of palliative guidelines and research has been on integrated care, aiming for close collaboration across secondary, primary, and home- and community-based care providers [8–11]. Informal caregivers might enable a patient to live at home longer in a pure practical manner, and in addition function as the patient’s advocate [12–16]. Informal caregivers therefore most likely influence the patients living situation by increasing their likelihood of living at home during their end-of-life, and thus decreasing their need for formal living (i.e., living in institutions). Informal caregivers are also likely to influence the level of end-of-life care that people receive in the different levels of the health care sector towards their end-of-life; both because the level of care that patients need depends on the patients living situation and on the informal caregivers ability to substitute formal care, in addition, the informal caregiver might ‘push the system’ to ensure that the patient receives what they perceive as sufficient amounts of care towards their end-of-life [11–13, 16–19]. Consequently, when evaluating the economics of end-of-life care, the focus should be on both formal care (in all levels of the sector) and informal care. However, due to limited access to data, most economic research of end-of-life care utilization and costs focus on single aspects of care, often hospital care, in selected cohorts (e.g. only for the elderly) [3, 20, 21]. In Norway, we have access to national registers with data on complete cohorts of patients. Cohorts can be linked at patient level to registers with information on the use and cost of health care services in all levels of the sector, and to registers with information on individual characteristics. This gives us a unique opportunity to give detailed descriptions of health care utilization and costs for non-selected cohorts of patients. In this paper, we employ Norwegian register data to increase the knowledge of end-of-life care, using colorectal cancer as an example.

Colorectal cancer is the 2nd most common cancer and the 3rd most common cause of cancer death worldwide [22, 23]. Because of the aging of the population, countries with decreasing age-adjusted incidence and mortality rates are also experiencing an increase in the absolute number of new cases and deaths from colorectal cancer [22, 24, 25]. Colorectal cancer will therefore continue to be a major health burden and cause of death in the future [25, 26]. While there is substantial economic evidence surrounding colorectal cancer population-based screenings, surgical procedures, and cytotoxic therapies, little economic evidence exists regarding the end-of-life treatment that colorectal cancer patients receive [27]. A spouse or a partner is generally the *primary source* of informal care [12, 18, 19]. Children are also a source of informal care, and studies have shown that the currently and previously married are more likely to have children, and thus also more likely to have access to informal care, compared to the never married [18, 19]. In colorectal cancer, research has shown that patients with a spouse or partner are less likely to present with metastatic cancer at diagnosis, are more likely to receive definitive therapy, and are less likely to die from their cancer [28, 29]. However, little is known about how having a spouse or a partner, or having children, affects colorectal cancer patients’ end-of-life.

The aim of this study was twofold; first, we wanted to describe the living situation and total health care utilization and costs at the end-of-life for an average patient dying from colorectal cancer. Second, we wanted to estimate the effect of marital status (as a proxy for availability of informal care from a spouse or a partner) on the living situation, health care utilization, and costs for patients dying from colorectal cancer after controlling for observed confounders guided by the Anderson model, which states that healthcare utilization depends on individuals’ predisposing and enabling characteristics, in addition to factors related to need for care [15].

## Methods

### Setting

#### The Norwegian health care system

In Norway, health care is publicly funded. There are few private providers and opting out of the public system is not an option. Hospitals are funded with block grants and activity-based funding via diagnosis-related groups (DRGs). Primary care services are funded by a combination of block grants and activity-based funding; i.e., GPs receive capitation payment, payment based on the type of healthcare service that they provide (through claims), in addition to patient co-payments. Home- and community-based healthcare services are paid for by block grants and patient co-payments; institutionalized care is primarily paid for by income-dependent out-of-pocket payments where patients pay up to 85% of their income for their long-term care [30]. All secondary or primary care providers who request reimbursement based on their activity through claims are responsible for providing continuous reports on their activity to either the Norwegian Health Economics Administration (HELFO) or the Norwegian Patient Register (NPR) [31, 32].

### Data sources and linkage

#### Study population

We identified all patients who died from colorectal cancer between 2009 and 2013 from the Norwegian Causes of Death Registry; i.e. all patients coded with colorectal cancer as their underlying cause of death (ICD 10 C18-C20) as noted by the physician who completed the death certificate [33]. The register covers 100% of the Norwegian population. All patients were linked to the Cancer Registry of Norway, where we obtained information on the type (ICD-10 coded) and time (between 1951 and 2013) of their primary cancer diagnosis [34]. This confirmed that all those dying from colorectal cancer were also coded with a colorectal cancer diagnosis in the Cancer Registry of Norway.

#### Secondary care services

NPR includes information on all claims from secondary care providers (hospitals) in Norway [32]. Each time a treatment is provided, a claim is sent to NPR. Claims include the patients’ diagnoses (ICD-10 codes) and the procedures provided. Based on this information, all hospital contacts are grouped into one of approximately 900 diagnosis-related groups (DRG) that reflect the type of treatment provided. Each DRG has an associated DRG cost, which is estimated as the mean cost reported from several hospitals that perform the relevant procedures. The DRG cost includes direct and indirect costs, the cost of complications while in hospital, and overhead costs, and is assumed to reflect the mean cost of the treatment provided, but excludes the cost of laboratory, radiology, and patients’ co-payments [35].

For each individual, we derived information from NPR on all treatments received in hospitals (grouped as inpatient treatments or outpatient treatments), the total number of days patients spent in the hospital, and the total cost of hospital treatments based on the DRG costs (see Table 1).

**Table 1:** Variables used to describe the patients living situation, health care utilization, and costs

	Variable	Units #	Source	Comments
Health care utilization				
**Secondary care**				
	Inpatient^a^	Treatment	NPR [32]	All inpatient treatments (including ‘day-treatment’ and ‘overnight-treatments’)
	Outpatient^a^	Treatment	NPR [32]	All outpatient treatments
**Primary care**				
	General practitioner	Consultations	KUHR [31]	One claim equals one consultation, including minor consultations such as telephone consultations and consultations for blood tests only
	Emergency room	Visits	KUHR [31]	One claim equals one visit
**Home- and community-based care**				
	Practical assistance	Hours	IPLOS [36]	Total number of hours
	Nursing assistance	Hours	IPLOS [36]	Total number of hours
**Living arrangements**				
	Hospital	Days	NPR [32]	Sum of days in hospital
	Long term institutions	Days	IPLOS [36]	Sum days in long term institutions
	Short term institutions	Days	IPLOS [36]	Sum days in short term institutions
	Home	Days	–	Sum days at home (= total number of days - days in hospital - days in long - short term institutions)
**Health care costs**				
Secondary care	Costs secondary care	NOK	NPR [32] KUHR [31]	Inpatient stay: DRG weight * unit costs. Outpatient stay: DRG weight * unit cost. Other costs not captured in DRG: laboratory, radiology and patient co-payment: (claim + co-payment) / 0,3
Primary care	Costs primary care	NOK	KUHR [31] HDIR [37]	Claim + out of pocket / 0,3
Home- and community-based care	Costs home- and community-based care	NOK	IPLOS [36] KOSTRA [38] Langeland [39]	Days in institution * NOK 2714 + hours of practical assistance * NOK 422 + hours of practical assistance * NOK 610
Total costs	Total costs	NOK	–	Sum of total costs secondary, primary and home- and community-based care

^a^The sum of inpatient and outpatient treatments, equals the total number of treatments in hospital

*NPR* Norwegian patient registry, *KUHR* The Control and Payment of Health Reimbursement register, *IPLOS* the Individual-based nursing and care statistics register, *HDIR* The Norwegian Directorate of Health, *KOSTRA* Municipality-State-Reporting

#### Primary care services

The Control and Payment of Health Reimbursement register (KUHR) includes information on all claims from primary care providers [31]. Each time a treatment is provided, a claim is sent to HELFO, and information is stored in the KUHR database. Claims include the patient’s diagnosis (ICD-10 codes), the treatment provided, and information on the patients’ co-payment. The type of treatment is coded according to current tariffs [40]. Each code in the tariffs has a cost attached to it, which reflects the mean cost of the treatment provided, excluding basic costs (provided through block grants) and patient co-payments.

We received information from KUHR on all claims from GP consultations and emergency room (ER) visits, and associated information on claim reimbursement and out-of-pocket payment per consultation/visit. In addition, we received information on claims from hospitals related to laboratory and radiology services, and all patient co-payments provided to hospitals (see Table 1).

#### Home- and community-based care

Home- and community-based services are not funded based on their activity and claims are therefore not gathered or reported. However, all municipalities are required to gather information on the number of patients who have applied for and/or received home and community-based care, with the goal of research, quality assurance, future planning and control of those services. This information is gathered in the individual-based nursing and care statistics register called IPLOS [36].

We gathered information from IPLOS on how many days patients lived in institutions, and whether the institution was considered short-term or long-term. We also gathered information on whether patients received home-based care (grouped as practical assistance or nursing assistance) and the magnitude of that care (total number of hours). The number of days that patients spent at home was estimated as the total number of days minus days in the hospital, days in a long-term institution and days in a short-term institution. We allowed for an overlap between hospital stays and institutional stays, since a patient’s place in an institution is not used by another patient if the patient is absent due to hospitalization (see Table 1).

#### Cost of care

To estimate the cost of secondary and primary care, we used information from NPR and KUHR on refund claims and patient co-payments (see Table 1). The costs of secondary care were estimated as 100% of the DRG cost. In a report, the Norwegian Directorate of Health estimated that the reimbursement claims and patient co-payments in KUHR summarized to approximately 30% of the total cost of treatments [37]. Based on this finding, we estimated the total cost of care from health care services included in the KUHR-database (GP consultations, ER visits, and radiology and laboratory services) by summarizing the total reimbursement and patient co-payments and dividing by 0.3, as has been done by previous researchers using the KUHR database [37, 41]. For home- and community-based care, we estimated the cost of institutionalized living by multiplying the number of days in an institution by the corrected gross operating expenses published by Statistics Norway (SSB) as a part of the Municipality-State-Reporting (KOSTRA) [38]. The cost of practical assistance and nursing assistance was estimated by multiplying the number of hours with practical assistance or nursing care, by the mean cost of care per hour as estimated by Langeland et al. [39], Table 1.

We estimated the costs in 2013 Norwegian Krone (NOK); in 2013 NOK 1 was approximately USD 0.17 or EURO 0.13.

#### Individual characteristics

SSB provided information on the populations’ marital status, age at death, gender, highest level of education, and income. Marital status six months prior to death was divided into those never married, married (or registered partner), or previously married (divorcee, widow/widower, or previously registered partner). Marital status was used as a proxy for availability of informal care.

Age was grouped as below 60 years, 60–69, 70–79, 80–89, or > 90. Education was grouped as primary school (0–10 years), secondary school (11–13 years), or higher education (> 14 years). Income was grouped in quartiles by gender for the entire cohort of patients dying (all dying in Norway) between 2008 and 2013. From NPR, we received information on the comorbidities of patients six months prior to death. Comorbidities were estimated and classified into the Charlson Comorbidity Index (CCI) based on hospital records (ICD-10 codes), including both the primary and secondary diagnoses, from 18 to 7 months before death [42, 43]. Comorbidities were grouped into mild/ moderate (0–4) or severe (> 5) comorbidities. Those who did not have a comorbidity recorded—because they did not have any contact with the hospital 18 to 7 months prior to their death—were assumed to have mild/moderate comorbidities.

### Ethics approval and consent to participate

The Norwegian Ethics Committee and the Norwegian Data Protection Authority, in addition to all the registry owners, approved this study. Registry owners gave us administrative permission to access and use the data. Registry owners include the Norwegian Directorate of health (NPR [32], KUHR [31], IPLOS [36]), the Cancer Registry of Norway [34], the National Institute of Public Health (the Norwegian Causes of Death Register [33]), and Statistics Norway (SSB, https://www.ssb.no/).

### Statistics

We used descriptive statistics to display sociodemographic and disease characteristics for the total population, and for the population according to marital status. Differences between groups (never married, married and previously married) were tested using chi-square statistics.

**Fig. 1:**
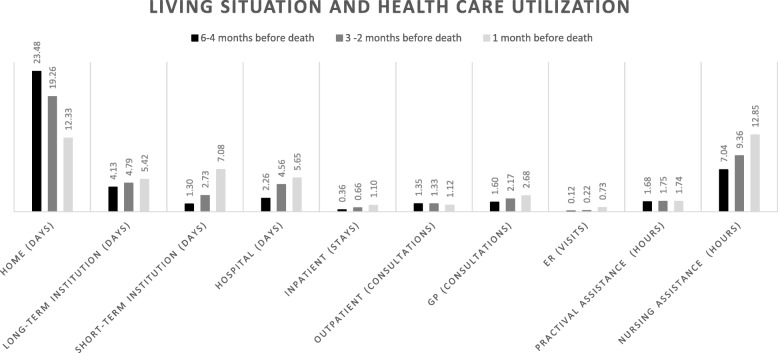
Living situation and health care utilization during the 3 periods 6–4 months, 3–2 months and 1 month before death. Numbers are given as the mean number (days, stays, consultations, visits or hours) per month during the periods

**Fig. 2:**
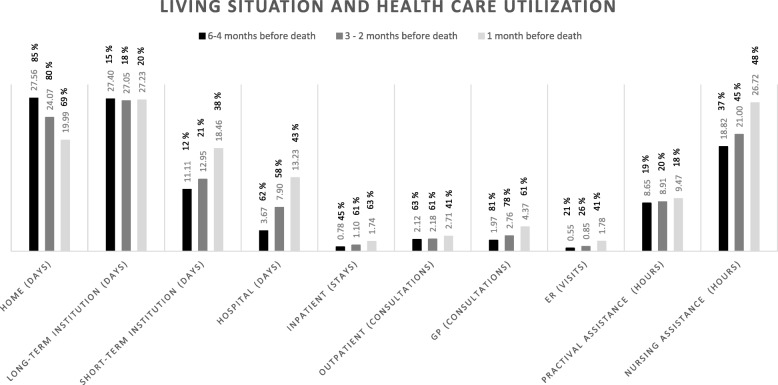
Living situation and health care utilization during the 3 periods 6–4 months, 3–2 months and 1 month before death. Numbers are given as the mean number (days, stays, consultations, visits or hours) per month during the periods for patients who used the different services (conditional use). The percentage of patients that used the service is displayed at the top of the bar chart

For all levels of health care use, we assumed that a missing registration meant no utilization. Information on health care use was provided to us in three periods: 6–4 months prior to death, 3–2 months prior to death, and 1 month prior to death. We defined the resource use of an average patient during the last six months of life as the mean use per month, per period (i.e. use during the 6–4 month period was divided by 3) (see Fig. 1). In a similar manner, we reported the length of stay conditioned on having at least one day at home or in an institution, and health care utilization conditioned on utilizing a service at least once (see Fig. 2). For tables with additional descriptive information corresponding to Fig. 1 and Fig. 2, see the Additional file 1: Table S1 and S2.

To estimate the effect difference on the living situation, health care utilization, and costs between the different groups of marital status, we ran separate multivariate regression models for all 14 outcome variables (all outcome variables described in Table 1). The regressions allow us to estimate the effect of marital status on the living situation, health care utilization, and costs for patients dying from colorectal cancer after controlling for observed confounders. Model selection was performed by first assessing the characteristics of the outcome variables, and then for each outcome variable, choosing the best model from groups of appropriate models. Models were selected using the Akaike information criterion (AIC) and the Bayesian information criterion (BIC) [44]. Variables on health care utilization and living arrangements were non-negatively skewed integer numbers (count variables), therefore, we chose between the Poisson, the Negative Binomial 1, and the Negative Binomial 2. Two of the outcome variables for living arrangements (days in a long-term institution and days at home) were neither Poisson nor Negative Binomially distributed, but had a large number of zeroes with a tail of increasing counts at higher values. For these two outcomes, we tested a two-part model using logistic regression in the first part and ordinary least square regressions in the second part (in addition to the Poisson, the Negative Binomial 1, and the Negative Binomial 2). The variables on costs were non-negatively skewed continuous numbers. We therefore ran generalized linear models (GLM), testing different links (identity, log, and power) and distributional families (Gaussian, inverse Gaussian, and Gamma). The model of choice for each outcome variable is reported together with the results. In all the multivariate models, we included covariates as defined in the section on ‘Individual characteristics.’ As a robustness check, we ran all our models with three different specifications of the Charlson Index: 1) in two groups (mild/ moderate or severe), 2) using the index as a continuous scale, and 3) using three groups of the index (mild (0–2), moderate (3–4) and severe (> 5)). Never-married patients were always the reference category. Unadjusted differences between the groups (never married, married, and previously married) were tested using univariate regression models. We used robust standard errors.

Results from the regression models are presented as the average marginal effects (AME) to enable interpretation on the variables’ original scale [45]. All regression analyses, including information on both AME and coefficients for all variables in the regression models, are displayed in the Additional file 1: Table S3, S4, S5, S6 and S7).

Analyses were performed in Stata 15.

## Results

### Descriptive statistics

A total of 7695 patients died of colorectal cancer in Norway between 2009 and 2013. The majority of those dying were between 80 and 89 years old, split evenly between males and females. Half of the patients died within two years after their diagnosis, 31% within the first year, and 19% during the second year. The majority (64%) of patients had mild/ moderate comorbidities six months before death.

Among the decedents, 3601 were married, 3359 were previously married, and 735 were never married. The individual characteristics differed across the groups (see Table 2).

**Table 2:** Descriptive statistics over the population dying from colorectal cancer in Norway between 2009 and 2013

	All		Never Married		Married		Previously Married		
	n = 7695		*n* = 735		*n* = 3601		*n* = 3359		
	No	%	No	%	No	%	No	%	*p*-values^a^
Age									
Below 60	797	(0.10)	192	(0.26)	441	(0.12)	164	(0.05)	***
60–69	1513	(0.20)	159	(0.22)	921	(0.26)	433	(0.13)	
70–79	2063	(0.27)	164	(0.22)	1166	(0.32)	733	(0.22)	
80–89	2502	(0.33)	160	(0.22)	942	(0.26)	1400	(0.42)	
Above 90	820	(0.11)	60	(0.08)	131	(0.04)	629	(0.19)	
Gender									
Female	3851	(0.50)	306	(0.42)	1314	(0.36)	2224	(0.66)	***
Education									
Primary school	3055	(0.41)	292	(0.45)	1154	(0.33)	1609	(0.48)	***
Secondary school	3344	(0.45)	269	(0.42)	1699	(0.49)	1376	(0.45)	
Higher education	1067	(0.14)	83	(0.13)	639	(0.18)	345	(0.10)	
Income									
Quartile 1	1551	(0.21)	169	(0.26)	871	(0.25)	511	(0.15)	***
Quartile 2	1679	(0.22)	150	(0.23)	627	(0.18)	902	(0.22)	
Quartile 3	1817	(0.24)	139	(0.22)	769	(0.22)	909	(0.27)	
Quartile 4	2419	(0.32)	186	(0.29)	1225	(0.35)	1008	(0.30)	
Charlson comorbidities									
Mild/ moderate	4946	(0.64)	457	(0.62)	2001	(0.56)	2488	(0.74)	***
Severe	2749	(0.36)	278	(0.38)	1600	(0.44)	871	(0.36)	
Years since diagnosis									
0–1 year	2364	(0.31)	239	(0.33)	1015	(0.28)	1110	(0.33)	***
1–2 years	1434	(0.19)	154	(0.21)	722	(0.20)	558	(0.17)	
2–3 years	875	(0.11)	81	(0.11)	439	(0.12)	355	(0.11)	
3–5 years	1321	(0.17)	125	(0.17)	651	(0.18)	545	(0.16)	
6–10 years	811	(0.11)	60	(0.08)	428	(0.12)	323	(0.10)	
More than 11 years	890	(0.12)	76	(0.00)	346	(0.00)	468	(0.00)	
Year of death									
2009	1514	(0.20)	148	(0.20)	719	(0.20)	647	(0.19)	n.e.
2010	1550	(0.20)	145	(0.20)	698	(0.19)	707	(0.20)	
2011	1515	(0.20)	151	(0.21)	686	(0.19)	678	(0.20)	
2012	1563	(0.20)	141	(0.19)	767	(0.21)	655	(0.20)	
2013	1553	(0.20)	150	(0.20)	731	(0.20)	672	(0.20)	

* = *p* < 0.05, ** = *p* < 0.01, *** = *p* < 0.001. n.e. = not estimated

^a^p-values estimated using Pearson’s chi square test between the marital status groups

Compared to patients who never married, married patients were older and were more frequently men. They had higher education and income, were less frequently newly diagnosed, and had more comorbidities. Compared to patients who never married, previously married patients were older, were more frequently women, and had fewer comorbidities. Otherwise, the never- and previously married were similar.

### Average patient

During the last six months of life, the average patient spent approximately four months at home, while he or she spent 27 days in a long-term institution, 16 days in a short-term institution and 21 days in the hospital. On average, patients utilized 3.5 patient stays, 7.8 outpatient consultations, 11.8 GP consultations, 1.5 ER visits, and received 10.2 h of practical assistance and 52.7 h of nursing assistance. The total healthcare costs during the last six months of life accumulated to NOK 399,340; whereof 58, 3 and 39% of the total costs were in the secondary, primary, and home- and community-based care level, respectively (see Table 3).

**Table 3:** Descriptive statistics of the health care utilization 6 months before death for all people dying from colorectal cancer, and divided between those never married, married and previously married. Numbers are presented as mean (SD), and *p*-values are based on univariate regressions (same regressions as used in Table 4, without controlling for covariates)

	All		Never married			Married			Previously married		
	n = 7695		n = 735			n = 3601			n = 3359		
	No	SD	No	SD		No	SD	*p*-values^a^	No	SD	*p*-values^a^
Living arrangements^b^											
Home	121.30	(60.54)	111.14	(64.41)	rc	139.49	(45.84)	***	104.03	(67.55)	**
Long term institutions	27.39	(61.33)	36.25	(68.41)	rc	10.11	(38.56)	***	43.97	(73.41)	**
Short term institutions	16.43	(30.15)	17.62	(31.42)	rc	12.55	(26.72)	***	20.32	(32.71)	*
Hospital	21.56	(19.60)	22.78	(21.20)	rc	23.65	(19.69)	***	19.06	(18.84)	***
Secondary care											
Inpatient stays	3.50	(4.09)	3.79	(5.59)	rc	3.93	(4.35)		2.96	(3.28)	***
Outpatient consultations	7.82	(9.57)	8.20	(10.85)	rc	10.14	(10.26)	***	5.25	(7.67)	***
Primary care											
General practitioner	11.81	(10.68)	10.64	(10.52)	rc	12.63	(10.83)	***	11.19	(10.48)	
Energency care contacts	1.53	(2.06)	1.51	(2.26)	rc	1.45	(2.07)		1.62	(2.01)	*
Home- and community-based care											
Practical assistance (hours)	10.27	(79.02)	37.05	(218.29)	rc	2.94	(28.70)	***	12.27	(52.83)	**
Nursing assistance (hours)	52.69	(133.73)	56.88	(121.76)	rc	44.32	(130.61)		60.74	(138.94)	
Costs											
Secondary care	232,447	(187,091)	245,246	(209,030)	rc	269,729	(190,046)	**	189,678	(169,036)	***
Primary care	11,553	(12,490)	10,790	(13,758)	rc	12,553	(13,675)	**	10,601	(10,653)	
Home- and community-based care	155,360	(205,652)	196,504	(239,295)	rc	89,773	(154,204)	***	216,669	(223,944)	*
Total costs	399,340	(233,946)	452,541	(279,106)	rc	372,055	(220,491)	***	416,948	(233,507)	**

^a^p-values estimated using univariate regression models. We used the same models as in the multivariate models (see Table 4)

^b^The sum of days is higher than 180 since patients can have a nursing home place, while staying in hospital for treatment

*rc* reference category

* = *p* < 0.05, ** = *p* < 0.01, *** = *p* < 0.001

As death approached, the average patient spent fewer days at home, and more days in the hospital, as well as and in long- and short-term institutions. There was an increase in health care utilization across all levels of the sector towards end-of-life, except for the number of outpatient consultations, which decreased (see Fig. 1). Towards end-of-life, costs increased more than twofold in all levels of the sector (see Additional file 1).

The closer the patients got to death (6–4, 3–2, and 1 months before death), the lower was the proportion of patients who spent any days at home (85, 80 and 69%). Those that did spend any days at home, spent fewer days at home as death approached. There was a slight increase in the percentage of patients who spent any days in a long-term institutions (15, 18, and 20%) and in their length of stay, while there was a larger increase in the number of patients who spent any days in a short-term institution (12, 21 and 38%) and in their length of stay. There was a decrease in the proportion of patients who spent any days in hospital (62, 58 and 43%), but the length of the hospital stay conditioned on having at least one day in hospital, increased (3.67, 7.90 and 13.23 days). The proportion of patients who had any inpatient stays or ER visits, and the proportion of patients who received any nursing assistance, increased towards the end-of-life, while there was a decrease in the number of patients who had any outpatient consultations or GP consultations, and in the number of patients who received any hours of practical assistance. For all types of health care utilization, use increased conditioned on utilizing a minimum of one day, visit, consultation, or hour of care (see Fig. 2).

Towards the end-of-life, conditional costs increased in all levels of the sector (see Additional file 1).

### Informal care

Table 3 displays descriptive statistics of the living situation, health care utilization and costs together with results from univariate analyses across the marital groups.

The multivariate analyses are reported in Table 4. In the Table, we display the predicted absolute numbers for the never married. For the married and previously married, we display the predicted difference to those never married, e.g. the predicted number of days the never married spent at home was 104.68, while the predicted difference in the number of days the married and previously married spent at home was 30.42 days and 7.06 days, respectively. Our results showed that, compared to the never married, married patients spent significantly fewer days in long-term (− 29 days) and short-term institutions (− 10 days), and significantly more days at home (30 days) and in the hospital (3 days). Married patients had significantly more outpatient treatments (3 treatments) and GP consultations (2 consultations), while they used significantly less practical assistance (− 9 h). Married patients had significantly more costly treatments in the secondary (NOK 41,535) and in the primary (NOK 2,051) health care level, while their costs in the home- and community-based care level were significantly lower (−NOK 137,604). Consequently, total costs were significantly lower for married patients (−NOK 65,621) compared to never married patents (see Table 4).

**Table 4:** Results of the regression analyses on health care utilization 6 months before death for all people dying from colorectal cancer, divided between those not married, married and previously married

	Never married	Married					Previously married					Model
	Reference value ^a^	Mean	*p*-values	SE	95% CI		Mean	*p*-values	SE	95% CI		
Living situation												
Home	104.68	30.42	***	(2.66)	25.19	35.64	7.06	*	(2.78)	1.61	12.51	Two part model (logistic regression/OLS)
Long-term institution	43.56	−29.16	***	(2.82)	−34.69	−23.62	−8.42	**	(2.95)	−14.20	−2.64	Two part model (logistic regression/OLS)
Short-term institution	23.04	−10.12	***	(1.90)	−13.85	−6.40	−3.65		(1.97)	−7.51	0.20	Negative Binomial 1
Hospital	19.23	2.90	***	(0.77)	1.39	4.41	1.21		(0.81)	−0.37	2.79	Negative Binomial 1
Health care utilization												
Inpatient stay	3.32	0.26		(0.21)	−0.15	0.67	−0.02		(0.20)	−0.41	0.37	Negative Binomial 2
Outpatient treatments	5.77	2.57	***	(0.27)	2.04	3.10	0.89	**	(0.27)	0.36	1.43	Negative Binomial 1
GP consultations	10.23	2.49	***	(0.40)	1.70	3.27	0.84	*	(0.41)	0.03	1.64	Negative Binomial 1
ER visits	1.52	−0.06		(0.07)	−0.20	0.09	0.09		(0.08)	−0.05	0.24	Negative Binomial 1
Hours home nursing	56.36	−6.59		(3.38)	−13.21	0.03	−0.52		(3.51)	−7.40	6.36	Negative Binomial 1
Hours practical assistance	13.17	−9.36	***	(1.21)	−11.73	−7.00	−1.39		(1.07)	−3.49	0.72	Negative Binomial 1
Costs												
Specialist health care	204,164	41,535	***	(8,746)	24,392	58,677	9,818		(8,339)	− 6,525	26,161	GLM (Identity/gamma)
Primary health care	9,727	2,051	***	(540)	992	3,109	303		(529)	− 733	1,340	GLM (Identity/gamma)
Home- and community-based care	241,005	− 137,604	***	(13,256)	− 163,586	−111,622	−50,943	***	(13,944)	−78,271	−23,611	GLM (Log/gamma)
Total costs	434,964	−65,621	***	(10,922)	−87,026	−44,215	−20,321		(11,279)	−42,430	1,788	GLM (Identity/gaussian)

The covariates age, gender, education, income, comorbidities and time since diagnosis was included in all analyses

^a^The predicted absolute values on their original scale (i.e. the model predicts that never married patients spend 104 days at home). These can be used to estimate the average value for the married and previously married. E.g. the predicted number of days Married patients spent at home was 135.09 (104.68 + 30.42)

* = *p* < 0.05, ** = *p* < 0.01, *** = *p* < 0.001

Multivariate analyses showed that, compared to the never married, previously married patients spend significantly more days at home (7 days), and significantly fewer days in long-term institutions (− 8 days). The previously married had significantly more outpatient treatments (0.89 treatments) and GP consultations (0.84 consultations) than the never married. Consequently, previously married patients had significantly lower costs in the home- and community-based care level (−NOK50,943). There was no difference in total costs between previously married and never married patients.

Results were robust for different specifications of the Charlson Index (see Additional file 1: Table S8).

## Discussion

This study gives a detailed description of the care that an unselected cohort of colorectal cancer patients receive at their end-of-life. We included all people dying of colorectal cancer in Norway between 2009 and 2013, and linked them to six national registers that included information on total health care utilization and total health care costs, in addition to sociodemographic characteristics such as marital status, which we used as a proxy for informal care. Our aims were twofold: first, we wanted to describe the living situation, total health care utilization, and costs at the end-of-life for an average patient dying from colorectal cancer. Second, we wanted to estimate how informal care (from a spouse or a partner) affects the living situation, health care utilization, and costs for patients dying from colorectal cancer. Our findings showed that towards the end-of-life, the average patient spends fewer days at home and more days in institutions (short-term institutions, long-term institutions and hospitals), and that resource use and costs increased in the secondary, primary, and home- and community-based care settings. Married patients’ living situations, health care utilization, and costs differed substantially from those who never married; they spent more days at home and in the hospital, and fewer days in institutions, while they utilized more health care services in all levels of the sector. Consequently, the costs were higher in the secondary and primary care level, but lower in the home- and community-based care level, for married compared to the never married. Similar but weaker patterns were found between the previously and never married.

Our findings of increased health care utilization and costs towards the end-of-life are in accordance with other studies [17, 20, 41, 46–48]. Our estimated costs for the last six months of life are high (~NOK 400,000), but plausible, when compared to cost estimates in other countries (ranging between NOK 150,000 – NOK 600,000) [17, 21, 47, 49]. Costs might vary because of differences in treatment and care across countries/studies, but also because of methodological differences, such as inclusion of different cost components (direct or total health care costs, costs in different levels of the sector), different time perspectives (6 months or 12 months, studies in the 1990s compared to studies now), and different cost levels [21, 46].

Other studies have also found that informal care (in particular marital status) plays an important role at patients’ end-of-life [17–19]. Farkilla et al. [17] found that informal care accounted for 33% of the total costs associated with colorectal cancer patients’ end-of-life care, but that those living with someone (cohabiting) had a non-significant (*p* = 0.08, *n* = 41) reduction in total costs, compared to those who lived alone [17]. Wachterman et al. [18] found that unmarried patients (dying from all causes) were less likely to receive informal care and more likely to live in nursing homes or in other institutions during their last year of life. Larsson et al. [19] found that the likelihood of receiving in-home help, for patients dying from all causes, was lower for those who were married than for those who were not married. They did not, however, find any differences in the likelihood of institutionalized living (during the last 5 years of life) or hospital care between those who were married and those who were not married (not distinguishing between the never- and previously married) [19].

Because of the descriptive nature of our study, we cannot say whether receiving more informal care (being married) is better than (yields higher quality care) receiving more formal care. This picture probably varies across dying patients, their informal caregivers and health care services, and is both complex and context-dependent. From the perspective of those who are dying, previous research has shown that people generally express a preference towards being cared for (and dying) at home at their end-of-life [5, 6]. Days spent at home has thus been acknowledged as a measurement of quality—a patient-centered goal and outcome—towards the end-of-life [50]. From the informal caregivers’ perspective, the negative effects of being an informal caregiver overrides the positive effects, regarding the informal caregiver’s physical and psychological health, employment status, and family relations [51, 52]. However, studies imply that the longer the duration of informal care, the more negative effects on the informal care giver [51]. Cancer patients need extensive care only for a limited time at their end of life [53, 54]. Consistent with this, research implies that home-based care yields positive effects for informal caregivers of cancer patients at their end-of-life [12]. Some studies find that care managers—who distribute the scarce resource of care among patients in municipalities—might discriminate against those that have access to informal care [55]. Under these circumstances, increased home-based care might actually have negative consequences for those who are dying and for the informal caregiver. Some in the health care sector perceive home-based care as cost saving [8]. However, home-based care is found to be positive for those who are dying as well as for informal caregivers when it is provided as comprehensive multidisciplinary palliative care [11, 12]. In comprehensive multidisciplinary palliative care, the decision as to where the patient ultimately receives care is based on the patient’s need and preferences and on the informal caregiver’s preferences. In addition, formal care providers have a high level of flexibility (e.g. on-call nursing and “back up” beds in case of emergency) so that those who stay at home can easily access high quality formal care [11, 12]. Studies find that the preference for place of care actually changes for a proportion of both patients and informal caregivers towards the very end of life; from home to institution [6]. Home-based care is not necessarily cost saving under these circumstances (in all levels of the sector), something we find support for in our analyses. While married patients spent more days at home and utilized fewer home- and community-based care services, on the other hand, they used more secondary and primary health care services.

Estimates of living situation, healthcare utilization and costs might vary among countries due to differences in the incidence and severity of diseases, in available resources (both informal and formal care), in clinical practice patterns, and in relative price levels [56]. Consequently, the results found in our analyses are most likely generalizable to countries with similarities to Norway; countries with a highly developed formal chronic care system, and with relatively small families sometimes dispersed over great distances.

This study has some limitations. We did not have insight into whether patients had any children. Previous studies have demonstrated that having a child also influences health care utilization at patients’ end-of-life [18, 19]. We observed that there was a difference between those who were never married and those who were previously married; this difference is possibly driven by the fact that the previously married are more likely to have children (and thus access to informal care) [18].

Similarly to others, we found patterns of substitution between formal and informal care [51]. However, we have no measure of actual use of informal care, but use marital status as a proxy for informal care. Thus, we cannot be sure of whether informal care has actually been provided to the patients, and further say whether informal care actually substitutes formal care. Previous studies indicate that informal care is a substitute for formal home care, while it is a complement for doctor and hospital visits [57]. This is the same pattern as we find; that those with access to informal care (married and previously married) use less home- and community based care services while utilizing more of secondary and primary health care services.

Despite our attempt to control for systematic differences between the cohabitating (married/ partners) and non-cohabitating (never- or previously married), confounding factors might still affect our results. We had access to a range of individual patient characteristics, and if using the framework of Andersen’s behavioral model [15], we had information both regarding patients’ predisposing characteristics (e.g., age and gender), enabling characteristics (e.g., education and income), and factors associated with need (e.g., comorbidities and time since diagnosis). Due to ethical considerations regarding data security, we were not able to get access to more precise information regarding patient’s types of comorbidities or activities of daily living. Knowing and controlling for these factors could make our estimates more precise and should be explored in greater detail in future research.

Our data did not reveal the patients’ place of death; however, this topic has been covered by several previous studies [21, 58]. Research shows that the preferred place of care does not always concur with the preferred place of death; as patients might want to be cared for at home while wishing to die in another institution (hospice/elderly care facility) [6]. Even though information on place of death would increase our understanding of health care utilization at the end-of-life, our contribution lies in the place and magnitude of care, rather than in the place of death.

Because of ethical considerations (linkage between two pseudonym registers), we did not include the cost of prescription drugs that patients received outside institutions. However, prescription drugs only account for a small percentage (1–2%) of the total costs in colorectal cancer care at their end-of-life [17, 41].

Also due to ethical considerations (anonymity), we did not have access to patients’ geographic location (municipality of residence or rural/urban living). In Norway, palliative care is structured differently across municipalities. The structure of palliative care affects whether patients who have informal caregivers can, in fact, be cared for in their homes at the end of their lives [10–12]. In the future, we hope that demographic information will be available so that we can explore the effect of variation across areas in more detail.

Lastly, readers of this paper should be aware that we estimated the costs of care in the secondary and primary health care setting using DRGs and tariffs; these estimates reflect how care providers are reimbursed and not the actual cost of care [35, 40]. Also, the cost of staying in a short- or long-term institution was assumed similar to the average cost of institutionalized living in Norway, estimates that might be imprecise. Micro-costing analyses on the actual cost of care should be performed in the future, and can be combined with our findings on health care utilization [59].

Despite these limitations, this comprehensive study can increase knowledge of actual resource allocations in a complete no selection population. Consequently, it can support political processes through educating, informing, and enlightening policy makers. Further, it can also be a valuable tool for care managers, at different levels of the health care sector, who are planning for future need for health care services. We think that care managers should be aware of the large differences in formal care provision between those with and without access to informal care. Our study can also be used as a stepping-stone for economic evaluations of care pathways for end of life care. For instance, based on our analysis we have shown that care at home has the potential of reducing the financial burden of the health care sector. However, in order to state whether care at home (informal or formal) provides a positive value to society, we should systematically evaluate all consequences of informal end-of-life care, and also include the alternative of extended formal care at home [60, 61]. To understand the complexity of end-of-life care, future economic analysis should incorporate both the costs and care burden of all formal and informal care givers, and the consequences of different types of care (i.e., consequence on patients and informal care givers quality of life).

## Conclusion

In this paper we described a complete population’s health care utilization and costs at all levels of the health care sector. We found that people dying from colorectal cancer spend 1/3 of their last six months in either institutions or hospitals, and that they utilize a substantial amount of health care services at all levels of the sector as they near the end of their life. We also estimated the effect of marital status on patients’ living situation, health care utilization, and total health care costs, and found that informal caregiving plays an important role at patients’ end-of-life. Our study thus highlights the importance of including both health care costs (in all levels of the sector) and societal costs (informal caregivers) in addition to quality indicators for patients and informal caregivers; the latter, in a long-term perspective, in cost-effectiveness studies in the future.

## Supplementary information

**Additional file 1: Table S1.** Descriptive statistics of the average patients’ resource use per month per period (e.g. the use in the 6–4 month period is estimated as the total use in the period divided by 3 months). Corresponds to Fig. 1 in the paper. **Table S2.** Descriptive statistics for the percentage of patients that use a resource, and their frequency of use. Resource use is displayed per month per period (e.g. the use in the 6–4 month period is estimated as the total use in the period divided by 3 months). Corresponds to Fig. 2 in the paper. **Table S3.** Regression analyses, displaying the average marginal effects, for colorectal cancer patients’ living situation 6 months prior to death. **Table S4**. Regression analyses, displaying the average marginal effects, for colorectal cancer patients’ health care utilization 6 months prior to death. **Table S5.** Regression analyses displaying the average marginal effects of the total cost of care in the different levels of the health care sector, for colorectal cancer patients’ 6 months prior to death. **Table S6.** Regression analyses, displaying the coefficients, for colorectal cancer patients’ living situation 6 months prior to death. **Table S7.** Regression analyses, displaying the coefficients, for colorectal cancer patients’ health care utilization 6 months prior to death. **Table S8.** Results when running our models with different specifications of the Charlson Comorbidity Index. Numbers presented as average marginal effects.

## Data Availability

The findings of this study are based on register data available from the Norwegian Directorate of health (NPR [32], KUHR [31], IPLOS [36]), the Cancer Registry of Norway [34], the National Institute of Public Health (the Norwegian Causes of Death Register [33]), and Statistics Norway (SSB, https://www.ssb.no/). The data used are covered by the permission granted for the current study, and are not publicly available.

## Abbreviations

AIC: Akaike information criterion
AME: Average marginal effects
BIC: Bayesian information criterion
CCI: Charlson comorbidity index
DRG: Diagnosis related groups
ER: Emergency room
GP: General practitioner
HELFO: Norwegian health economics administration
ICD: International classification of diseases
IPLOS: Individual-based nursing and care statistics register
KOSTRA: Municipality State Reporting
KUHR: The control and payment of health reimbursement register
NOK: Norwegian krone
NPR: Norwegian patient registry
SSB: Statistics Norway
USD: United States dollars
