# Supplementary material for: Till death do us part: the effect of marital status on health care utilization and costs at end-of-life. A register study on all colorectal cancer decedents in Norway between 2009 and 2013
Source: BMC Health Serv Res. 2020 Feb 13;20:115. doi: 10.1186/s12913-019-4794-6 (PMC7020544; doi:10.1186/s12913-019-4794-6)
Supplement: Supplementary file 1 — Additional file 1: Table S1. Descriptive statistics of the average patients’ resource use per month per period (e.g. the use in the 6–4 month period is estimated as the total use in the period divided by 3 months). Corresponds to Fig. 1 in the paper. Table S2. Descriptive statistics for the percentage of patients that use a resource, and their frequency of use. Resource use is displayed per month per period (e.g. the use in the 6–4 month period is estimated as the total use in the period divided by 3 months). Corresponds to Fig. 2 in the paper. Table S3. Regression analyses, displaying the average marginal effects, for colorectal cancer patients’ living situation 6 months prior to death. Table S4. Regression analyses, displaying the average marginal effects, for colorectal cancer patients’ health care utilization 6 months prior to death. Table S5. Regression analyses displaying the average marginal effects of the total cost of care in the different levels of the health care sector, for colorectal cancer patients’ 6 months prior to death. Table S6. Regression analyses, displaying the coefficients, for colorectal cancer patients’ living situation 6 months prior to death. Table S7. Regression analyses, displaying the coefficients, for colorectal cancer patients’ health care utilization 6 months prior to death. Table S8. Results when running our models with different specifications of the Charlson Comorbidity Index. Numbers presented as average marginal effects. [file 12913_2019_4794_MOESM1_ESM.docx]

**Additional file 1**

Table S1: Descriptive statistics of the average patients’ resource use per month per period (e.g. the use in the 6-4 month period is estimated as the total use in the period divided by 3 months). Corresponds to Figure 1 in the paper.

|  | **4-6 months before death** | | | | | **2 - 3 months before death** | | | | | **1 month before death** | | | | | **Total 6 months before death** | | | | |
| --- | --- | --- | --- | --- | --- | --- | --- | --- | --- | --- | --- | --- | --- | --- | --- | --- | --- | --- | --- | --- |
|  | Mean | SD | 50 % | 75 % | 99 % | Mean | SD | 50 % | 75 % | 99 % | Mean | SD | 50 % | 75 % | 99 % | Mean | SD | 50 % | 75 % | 99 % |
|  |  |  |  |  |  |  |  |  |  |  |  |  |  |  |  |  |  |  |  |  |
| **Living arrangements** |  |  |  |  |  |  |  |  |  |  |  |  |  |  |  |  |  |  |  |  |
| Home | 23.48 | 11.08 | 29.67 | 30.67 | 30.67 | 19.26 | 12.06 | 24.00 | 31.00 | 31.00 | 12.33 | 11.39 | 11.00 | 23.00 | 31.00 | 121.30 | 60.54 | 145.00 | 166.00 | 185.00 |
| Long-term institution | 4.13 | 10.23 | 0.00 | 0.00 | 30.67 | 4.79 | 12.37 | 0.00 | 0.00 | 31.00 | 5.42 | 11.43 | 0.00 | 0.00 | 31.00 | 27.39 | 61.33 | 0.00 | 0.00 | 185.00 |
| Short-term institution | 1.30 | 4.73 | 0.00 | 0.00 | 30.67 | 2.73 | 6.85 | 0.00 | 0.00 | 31.00 | 7.08 | 10.99 | 0.00 | 12.00 | 31.00 | 16.43 | 30.15 | 0.00 | 22.00 | 162.00 |
| Hospital | 2.26 | 2.63 | 1.33 | 4.00 | 9.67 | 4.56 | 6.49 | 1.50 | 7.00 | 29.00 | 5.65 | 10.84 | 0.00 | 7.00 | 50.00 | 21.57 | 19.61 | 17.00 | 30.00 | 90.00 |
| **Secondary care** |  |  |  |  |  |  |  |  |  |  |  |  |  |  |  |  |  |  |  |  |
| Inpatient stays | 0.36 | 0.72 | 0.00 | 0.33 | 2.67 | 0.66 | 0.94 | 0.50 | 1.00 | 3.50 | 1.10 | 1.31 | 1.00 | 2.00 | 5.00 | 3.50 | 4.09 | 3.00 | 5.00 | 15.00 |
| Outpatient consultations | 1.35 | 1.89 | 0.33 | 2.00 | 8.00 | 1.33 | 2.00 | 0.50 | 2.00 | 9.00 | 1.12 | 2.27 | 0.00 | 1.00 | 12.00 | 7.82 | 9.57 | 4.00 | 12.00 | 39.00 |
| **Primary care** |  |  |  |  |  |  |  |  |  |  |  |  |  |  |  |  |  |  |  |  |
| General practitioner (no of contacts) | 1.60 | 1.67 | 1.33 | 2.33 | 7.33 | 2.17 | 2.34 | 1.50 | 3.00 | 10.50 | 2.68 | 3.97 | 1.00 | 4.00 | 18.00 | 11.81 | 10.68 | 9.00 | 16.00 | 49.00 |
| Physician at municipal emergency room (no of contacts) | 0.12 | 0.28 | 0.00 | 0.00 | 1.33 | 0.22 | 0.48 | 0.00 | 0.50 | 2.00 | 0.73 | 1.22 | 0.00 | 1.00 | 5.00 | 1.53 | 2.06 | 1.00 | 2.00 | 9.00 |
| **Home- and community-based care** |  |  |  |  |  |  |  |  |  |  |  |  |  |  |  |  |  |  |  |  |
| Practical assistance (no of hours) | 1.68 | 13.14 | 0.00 | 0.00 | 22.86 | 1.75 | 13.72 | 0.00 | 0.00 | 24.36 | 1.74 | 13.37 | 0.00 | 0.00 | 26.57 | 10.27 | 79.02 | 0.00 | 0.00 | 125.53 |
| Nursing assistance (no of hours) | 7.04 | 21.50 | 0.00 | 4.38 | 92.00 | 9.36 | 24.76 | 0.00 | 8.36 | 110.71 | 12.85 | 32.47 | 0.19 | 13.50 | 131.00 | 52.69 | 133.72 | 6.75 | 52.50 | 565.57 |
| **Costs** |  |  |  |  |  |  |  |  |  |  |  |  |  |  |  |  |  |  |  |  |
| Secondary care | 24,410 | 33,223 | 8,444 | 39,185 | 136,396 | 43,462 | 55,773 | 28,427 | 67,063 | 207,172 | 72,291 | 940,002 | 56,826 | 106,388 | 306,018 | 232,446 | 187,090 | 203,228 | 326,778 | 896,736 |
| Primary care | 1,400 | 1,811 | ,889 | 1,922 | 8,565 | 1,951 | 2,687 | 1,202 | 2,636 | 11,994 | 3,430 | 5,807 | 1,430 | 4,326 | 27,827 | 11,532 | 12,490 | 8,338 | 15,041 | 57,674 |
| Home- and community-based care | 19,724 | 34,662 | ,924 | 19,883 | 120,363 | 26,846 | 37,745 | 4,913 | 48,705 | 129,487 | 42,494 | 41,833 | 30,321 | 84,119 | 143,754 | 155,359 | 205,652 | 57,941 | 228,454 | 738,317 |
| Total costsᵈ | 45,535 | 44,768 | 33,138 | 82,236 | 179,600 | 72,259 | 61,355 | 68,578 | 101,442 | 251,661 | 118,215 | 88,704 | 95,215 | 149,970 | 358,755 | 399,339 | 233,945 | 362,454 | 524,442 | 1126,919 |

Table S2: Descriptive statistics for the percentage of patients that use a resource, and their frequency of use. Resource use is displayed per month per period (e.g. the use in the 6-4 month period is estimated as the total use in the period divided by 3 months). Corresponds to Figure 2 in the paper.

|  |  | 4-6 months before death | | | | |  | 2 - 3 months before death | | | | |  | 1 month before death | | | | |  | Total 6 months before death | | | | |
| --- | --- | --- | --- | --- | --- | --- | --- | --- | --- | --- | --- | --- | --- | --- | --- | --- | --- | --- | --- | --- | --- | --- | --- | --- |
|  | (%) ^1^ | Mean | SD | 50 % | 75 % | 99 % | (%)^1^ | Mean | SD | 50 % | 75 % | 99 % | (%)^1^ | Mean | SD | 50 % | 75 % | 99 % | (%)^1^ | Mean | SD | 50 % | 75 % | 99 % |
| **Living arrangements** |  |  |  |  |  |  |  |  |  |  |  |  |  |  |  |  |  |  |  |  |  |  |  |  |
| Home | (0.85) | 27.56 | 5.63 | 30.67 | 30.67 | 30.67 | (0.80) | 24.07 | 8.14 | 27.00 | 31.00 | 31.00 | (0.69) | 19.99 | 9.35 | 19.00 | 26.00 | 31.00 | (0.86) | 141.15 | 38.26 | 152.00 | 169.00 | 185.00 |
| Long-term institution | (0.15) | 27.40 | 7.51 | 30.67 | 30.67 | 30.67 | (0.18) | 27.05 | 8.10 | 31.00 | 31.00 | 31.00 | (0.20) | 27.23 | 7.89 | 31.00 | 31.00 | 31.00 | (0.21) | 130.49 | 66.84 | 173.00 | 185.00 | 185.00 |
| Short-term institution | (0.12) | 11.11 | 9.11 | 8.00 | 15.00 | 30.67 | (0.21) | 12.95 | 9.50 | 10.50 | 19.00 | 31.00 | (0.38) | 18.46 | 10.22 | 19.00 | 31.00 | 31.00 | (0.46) | 36.01 | 35.88 | 24.00 | 48.00 | 185.00 |
| Hospital | (0.62) | 3.67 | 2.47 | 3.33 | 5.33 | 10.00 | (0.58) | 7.90 | 6.82 | 6.00 | 11.00 | 31.00 | (0.43) | 13.23 | 13.23 | 9.00 | 18.00 | 64.00 | (0.90) | 23.96 | 19.23 | 19.00 | 33.00 | 92.00 |
| **Secondary care** |  |  |  |  |  |  |  |  |  |  |  |  |  |  |  |  |  |  |  |  |  |  |  |  |
| Inpatient stays | (0.46) | 0.78 | 0.90 | 0.67 | 1.00 | 3.67 | (0.61) | 1.10 | 0.99 | 1.00 | 1.50 | 4.00 | (0.63) | 1.74 | 1.27 | 1.00 | 2.00 | 5.00 | (0.91) | 3.85 | 4.13 | 3.00 | 5.00 | 15.00 |
| Outpatient consultations | (0.64) | 2.12 | 2.00 | 1.33 | 3.33 | 8.67 | (0.61) | 2.18 | 2.17 | 1.50 | 3.00 | 10.50 | (0.41) | 2.71 | 2.86 | 2.00 | 3.00 | 16.00 | (0.80) | 9774.00 | 9.76 | 6.00 | 15.00 | 41.00 |
| **Primary care** |  |  |  |  |  |  |  |  |  |  |  |  |  |  |  |  |  |  |  |  |  |  |  |  |
| General practitioner (no of contacts) | (0.81) | 1.97 | 1.65 | 1.67 | 2.67 | 7.67 | (0.78) | 2.76 | 2.31 | 2.00 | 3.50 | 11.50 | (0.61) | 4.37 | 4.27 | 3.00 | 6.00 | 20.00 | (0.92) | 12.90 | 10.51 | 10.00 | 18.00 | 49.00 |
| Physician at municipal emergency room (no of contacts) | (0.21) | 0.55 | 0.38 | 0.33 | 0.67 | 2.00 | (0.26) | 0.85 | 0.60 | 0.50 | 1.00 | 3.00 | (0.41) | 1.78 | 1.32 | 1.00 | 2.00 | 7.00 | (0.62) | 2.48 | 2.13 | 2.00 | 3.00 | 10.00 |
| **Home and community based care** |  |  |  |  |  |  |  |  |  |  |  |  |  |  |  |  |  |  |  |  |  |  |  |  |
| Practical assistance (no of hours) | (0.19) | 8.65 | 28.83 | 3.29 | 5.47 | 143.67 | (0.2) | 8.91 | 29.95 | 3.32 | 5.54 | 145.00 | (0.18) | 9.47 | 30.06 | 3.57 | 5.89 | 132.85 | (0.24) | 43.24 | 157.76 | 18.26 | 26.42 | 635.00 |
| Nursing assistance (no of hours) | (0.37) | 18.82 | 31.86 | 8.76 | 22.17 | 138.33 | (0.45) | 21.00 | 33.64 | 10.32 | 25.82 | 150.50 | (0.48) | 26.72 | 42.72 | 14.74 | 31.25 | 190.42 | (0.57) | 92.25 | 166.34 | 41.04 | 105.71 | 740.00 |
| **Costs** |  |  |  |  |  |  |  |  |  |  |  |  |  |  |  |  |  |  |  |  |  |  |  |  |
| Secondary care | (0.84) | 29,077 | 34,338 | 18,222 | 46,564 | 141,363 | (0.88) | 49,540 | 56,961 | 33,560 | 75,619 | 212,508 | (0.80) | 90,762 | 97,045 | 77,068 | 128,141 | 320,565 | (0.98) | 236,378 | 186,186 | 206,313 | 330,041 | 900,379 |
| Primary care | (0.84) | 1,675 | 1,860 | 1,175 | 2,198 | 9,063 | (0.82) | 2,370 | 2,788 | 1,614 | 3,027 | 12,561 | (0.74) | 4,617 | 6,318 | 2,689 | 5,574 | 29,831 | (0.96) | 12,056 | 12,521 | 8,734 | 15,539 | 58,848 |
| Home- and community-based care | (0.55) | 35,856 | 40,072 | 15,047 | 83,215 | 140,360 | (0.67) | 40,019 | 39,958 | 23,065 | 84,120 | 144,122 | (0.80) | 53,247 | 40,253 | 55,147 | 84,119 | 151,520 | (0.81) | 191,095 | 212,585 | 97,691 | 320,779 | 772,460 |
| Total costsᵈ | (0.97) | 47,184 | 44,710 | 35,539 | 83,215 | 180,636 | (0.99) | 73,269 | 61,181 | 70,159 | 102,068 | 251,987 | (0.97) | 117,777 | 8,688 | 95,076 | 148,897 | 355,780 | (1.00) | 398,258 | 233,848 | 362,670 | 525,491 | 1126,919 |

^1^ Percentage of the total population (n=7,695)

Table S3: Regression analyses, displaying the average marginal effects, for colorectal cancer patients’ living situation 6 months prior to death

|  | Home | | | | Long-term institution | | | | Short term institution | | | | Hospital | | | |
| --- | --- | --- | --- | --- | --- | --- | --- | --- | --- | --- | --- | --- | --- | --- | --- | --- |
|  | Margins (AME) |  | Confidence interval | | Margins (AME) |  | Confidence interval | | Margins (AME) |  | Confidence interval | | Margins (AME) |  | Confidence interval | |
| **Marital status** |  |  |  |  |  |  |  |  |  |  |  |  |  |  |  |  |
| Never married | r.c. |  | - | - | r.c. |  | - | - | r.c. |  | - | - | r.c. |  | - | - |
| Married | 30.42 | *** | 25.19 | 35.64 | -29.16 | *** | -34.69 | -23.62 | -10.12 | *** | -13.85 | -6.40 | 2.90 | *** | 1.39 | 4.41 |
| Previously married | 7.06 | * | 1.61 | 12.51 | -8.42 | ** | -14.20 | -2.64 | -3.65 |  | -7.51 | 0.20 | 1.21 |  | -0.37 | 2.79 |
| **Age** |  |  |  |  |  |  |  |  |  |  |  |  |  |  |  |  |
| Below 60 | r.c. |  | - | - | r.c. |  | - | - | r.c. |  | - | - | r.c. |  | - | - |
| 60-69 | 0.26 |  | -4.44 | 4.97 | 1.55 |  | -2.63 | 5.73 | 4.14 | *** | 2.15 | 6.13 | -3.99 | *** | -6.12 | -1.86 |
| 70-79 | -8.29 | ** | -12.97 | -3.62 | 9.11 | *** | 4.92 | 13.30 | 10.37 | *** | 8.33 | 12.41 | -6.83 | *** | -8.96 | -4.70 |
| 80-89 | -20.21 | *** | -25.10 | -15.32 | 24.28 | *** | 19.90 | 28.66 | 14.86 | *** | 12.79 | 16.93 | -13.77 | *** | -15.90 | -11.64 |
| Above 90 | -40.22 | *** | -46.95 | -33.49 | 48.70 | *** | 42.24 | 55.16 | 12.53 | *** | 9.76 | 15.30 | -20.58 | *** | -22.80 | -18.36 |
| **Gender** |  |  |  |  |  |  |  |  |  |  |  |  |  |  |  |  |
| Men | r.c. |  | - | - | r.c. |  | - | - | r.c. |  | - | - | r.c. |  | - | - |
| Women | -5.59 | *** | -8.28 | -2.89 | 1.93 |  | -0.76 | 4.63 | 2.19 | ** | 0.64 | 3.74 | 0.08 |  | -0.80 | 0.97 |
| **Education** |  |  |  |  |  |  |  |  |  |  |  |  |  |  |  |  |
| Primary school | r.c. |  | - | - | r.c. |  | - | - | r.c. |  | - | - | r.c. |  | - | - |
| High school (14 years) | 2.44 |  | -0.40 | 5.28 | -2.72 |  | -5.49 | 0.05 | 0.61 |  | -1.06 | 2.28 | 1.15 | * | 0.18 | 2.13 |
| Higher education | 5.30 | * | 0.59 | 10.02 | -6.24 | * | -11.02 | -1.46 | -0.38 |  | -3.09 | 2.32 | 0.68 |  | -0.74 | 2.09 |
| **Income** |  |  |  |  |  |  |  |  |  |  |  |  |  |  |  |  |
| 0% - 24% | r.c. |  | - | - | r.c. |  | - | - | r.c. |  | - | - | r.c. |  | - | - |
| 25% - 49% | 3.20 |  | -0.81 | 7.21 | -3.66 |  | -7.55 | 0.22 | -0.67 |  | -2.93 | 1.59 | 1.03 |  | -0.29 | 2.35 |
| 50 % -74% | 4.77 | * | 0.76 | 8.79 | -6.68 | ** | -10.66 | -2.71 | 0.08 |  | -2.24 | 2.40 | 1.26 |  | -0.05 | 2.56 |
| 75% - 100% | 10.61 | *** | 6.52 | 14.71 | -13.25 | *** | -17.38 | -9.13 | -2.46 | * | -4.75 | -0.17 | 2.37 | *** | 1.03 | 3.70 |
| **Comorbidity** |  |  |  |  |  |  |  |  |  |  |  |  |  |  |  |  |
| Mild/ moderate | r.c. |  | - | - | r.c. |  | - | - | r.c. |  | - | - | r.c. |  | - | - |
| Severe | 11.94 | *** | 8.92 | 14.96 | -14.09 | *** | -17.03 | -11.15 | 3.54 | *** | 1.55 | 5.52 | 0.80 |  | -0.22 | 1.82 |
| **Years since diagnosis** |  |  |  |  |  |  |  |  |  |  |  |  |  |  |  |  |
| 0-1 | r.c. |  | - | - | r.c. |  | - | - | r.c. |  | - | - | r.c. |  | - | - |
| 1-2 | -5.09 | ** | -8.85 | -1.33 | 5.56 | ** | 1.82 | 9.30 | 0.27 |  | -2.03 | 2.56 | -3.87 | *** | -5.21 | -2.52 |
| 2-3 | -9.60 | *** | -14.36 | -4.83 | 9.37 | *** | 4.56 | 14.18 | 2.46 |  | -0.38 | 5.30 | -6.17 | *** | -7.66 | -4.67 |
| 3-5 | -8.40 | *** | -12.40 | -4.39 | 9.97 | *** | 6.04 | 13.90 | 0.42 |  | -2.02 | 2.85 | -5.67 | *** | -7.00 | -4.34 |
| 6-10 | -12.46 | *** | -17.11 | -7.82 | 13.48 | *** | 8.86 | 18.10 | 1.27 |  | -1.55 | 4.09 | -5.50 | *** | -7.02 | -3.98 |
| 11 + | -10.49 | *** | -14.81 | -6.17 | 11.35 | *** | 7.36 | 15.33 | -0.15 |  | -2.52 | 2.22 | -4.47 | *** | -6.08 | -2.87 |
| **Model** | *Two part model (logistic regression/ OLS)* | | | | *Two part model (logistic regression/ OLS)* | | | | *Negative Binomial 1* | | | | *Negative Binomial 1* | | | |
| Alfa/ Delta** |  |  |  |  | *533.87* |  | *510.63* | *558.17* | *6.53* |  | *6.26* | *6.81* | *18.44* |  | *17.69* | *19.21* |
| AIC | *69533* |  |  |  | *24160* |  |  |  | *43670* |  |  |  | *59585* |  |  |  |
| BIC | *69796* |  |  |  | *24423* |  |  |  | *43808* |  |  |  | *59723* |  |  |  |

*** = p < 0.05, ** = p < 0.01, *** = p < 0.001**

**r.c. = reference category**

Table S4: Regression analyses, displaying the average marginal effects, for colorectal cancer patients’ health care utilization 6 months prior to death

|  | Inpatient stays | | | | Outpatient | | | | GP | | | | ER | | | | Practical assistance (hours) | | | | Home nursing | | | |
| --- | --- | --- | --- | --- | --- | --- | --- | --- | --- | --- | --- | --- | --- | --- | --- | --- | --- | --- | --- | --- | --- | --- | --- | --- |
|  | Margins (AME) | | Confidence interval | | Margins (AME) | | Confidence interval | | Margins (AME) | | Confidence interval | | Margins (AME) | | Confidence interval | | Margins (AME) | | Confidence interval | | Margins (AME) | | Confidence interval | |
| **Marital status** |  |  |  |  |  |  |  |  |  |  |  |  |  |  |  |  |  |  |  |  |  |  |  |  |
| Never married | r.c. |  | - | - | r.c. |  | - | - | r.c. |  | - | - | r.c. |  | - | - | r.c. |  | - | - | r.c. |  | - | - |
| Married | 0.26 |  | -0.15 | 0.67 | 2.57 | *** | 2.04 | 3.10 | 2.49 | *** | 1.70 | 3.27 | -0.06 |  | -0.20 | 0.09 | -9.36 | *** | -11.73 | -7.00 | -6.59 |  | -13.21 | 0.03 |
| Previously married | -0.02 |  | -0.41 | 0.37 | 0.89 | ** | 0.36 | 1.43 | 0.84 | * | 0.03 | 1.64 | 0.09 |  | -0.05 | 0.24 | -1.39 |  | -3.49 | 0.72 | -0.52 |  | -7.40 | 6.36 |
| **Age** |  |  |  |  |  |  |  |  |  |  |  |  |  |  |  |  |  |  |  |  |  |  |  |  |
| Below 60 | r.c. |  | - | - | r.c. |  | - | - | r.c. |  | - | - | r.c. |  | - | - | r.c. |  | - | - | r.c. |  | - | - |
| 60-69 | -0.32 |  | -0.73 | 0.09 | -2.13 | *** | -2.95 | -1.31 | 0.06 |  | -0.78 | 0.91 | 0.02 |  | -0.14 | 0.18 | -0.25 |  | -1.62 | 1.11 | 3.38 |  | -2.24 | 9.00 |
| 70-79 | -0.98 | *** | -1.38 | -0.59 | -4.30 | *** | -5.12 | -3.48 | 1.00 | * | 0.15 | 1.85 | 0.22 | ** | 0.05 | 0.38 | 1.98 | ** | 0.58 | 3.37 | 6.62 | * | 1.02 | 12.21 |
| 80-89 | -1.93 | *** | -2.33 | -1.54 | -7.79 | *** | -8.58 | -6.99 | 0.77 |  | -0.12 | 1.66 | 0.30 | *** | 0.14 | 0.47 | 5.49 | *** | 3.93 | 7.05 | 22.77 | *** | 16.47 | 29.07 |
| Above 90 | -2.87 | *** | -3.27 | -2.47 | -9.54 | *** | -10.35 | -8.73 | -2.21 | *** | -3.30 | -1.11 | 0.17 |  | -0.03 | 0.37 | 6.67 | *** | 4.60 | 8.75 | 18.39 | *** | 9.83 | 26.94 |
| **Gender** | 0.00 |  | 0.00 | 0.00 | 0.00 |  | 0.00 | 0.00 | 0.00 |  | 0.00 | 0.00 | 0.00 |  | 0.00 | 0.00 | 0.00 |  | 0.00 | 0.00 | 0.00 |  | 0.00 | 0.00 |
| Men | 0.00 |  | 0.00 | 0.00 | 0.00 |  | 0.00 | 0.00 | 0.00 |  | 0.00 | 0.00 | 0.00 |  | 0.00 | 0.00 | 0.00 |  | 0.00 | 0.00 | 0.00 |  | 0.00 | 0.00 |
| Women | -0.07 |  | -0.25 | 0.10 | -0.30 |  | -0.63 | 0.02 | -0.14 |  | -0.61 | 0.33 | -0.11 | * | -0.19 | -0.02 | 4.70 | *** | 3.68 | 5.72 | 7.38 | *** | 3.88 | 10.88 |
| **Education** |  |  |  |  |  |  |  |  |  |  |  |  |  |  |  |  |  |  |  |  |  |  |  |  |
| Primary school | r.c. |  | - | - | r.c. |  | - | - | r.c. |  | - | - | r.c. |  | - | - | r.c. |  | - | - | r.c. |  | - | - |
| High school (14 years) | 0.13 |  | -0.07 | 0.33 | 0.76 | *** | 0.42 | 1.11 | -0.63 | * | -1.15 | -0.12 | -0.20 | *** | -0.29 | -0.11 | 0.79 |  | -0.15 | 1.72 | -0.54 |  | -4.29 | 3.22 |
| Higher education | 0.18 |  | -0.13 | 0.50 | 1.42 | *** | 0.88 | 1.95 | -1.09 | ** | -1.83 | -0.35 | -0.31 | *** | -0.44 | -0.17 | 1.57 |  | -0.16 | 3.31 | -0.49 |  | -6.16 | 5.17 |
| **Income** |  |  |  |  |  |  |  |  |  |  |  |  |  |  |  |  |  |  |  |  |  |  |  |  |
| 0% - 24% | r.c. |  | - | - | r.c. |  | - | - | r.c. |  | - | - | r.c. |  | - | - | r.c. |  | - | - | r.c. |  | - | - |
| 25% - 49% | -0.04 |  | -0.36 | 0.28 | 0.33 |  | -0.13 | 0.80 | 0.95 | ** | 0.25 | 1.65 | -0.10 |  | -0.22 | 0.02 | -0.39 |  | -1.73 | 0.95 | 3.76 |  | -1.42 | 8.94 |
| 50 % -74% | -0.03 |  | -0.31 | 0.26 | 1.08 | *** | 0.62 | 1.54 | 1.29 | *** | 0.61 | 1.97 | -0.09 |  | -0.22 | 0.03 | -0.11 |  | -1.46 | 1.24 | 3.84 |  | -1.22 | 8.90 |
| 75% - 100% | 0.08 |  | -0.22 | 0.38 | 1.84 | *** | 1.37 | 2.32 | 0.89 | * | 0.20 | 1.59 | -0.25 | *** | -0.38 | -0.13 | -2.04 | ** | -3.42 | -0.65 | 0.18 |  | -4.93 | 5.30 |
| **Comorbidity** |  |  |  |  |  |  |  |  |  |  |  |  |  |  |  |  |  |  |  |  |  |  |  |  |
| Mild/ moderate | r.c. |  | - | - | r.c. |  | - | - | r.c. |  | - | - | r.c. |  | - | - | r.c. |  | - | - | r.c. |  | - | - |
| Severe | 0.52 | *** | 0.27 | 0.77 | 3.71 | *** | 3.32 | 4.10 | 1.99 | *** | 1.43 | 2.56 | -0.02 |  | -0.12 | 0.08 | 2.04 | ** | 0.82 | 3.25 | 27.94 | *** | 23.13 | 32.74 |
| **Years since diagnosis** |  |  |  |  |  |  |  |  |  |  |  |  |  |  |  |  |  |  |  |  |  |  |  |  |
| 0-1 | r.c. |  | - | - | r.c. |  | - | - | r.c. |  | - | - | r.c. |  | - | - | r.c. |  | - | - | r.c. |  | - | - |
| 1-2 | -0.08 |  | -0.28 | 0.13 | 1.09 | *** | 0.60 | 1.58 | -0.32 |  | -1.01 | 0.37 | 0.01 |  | -0.11 | 0.13 | -0.39 |  | -1.68 | 0.90 | 5.65 | * | 0.48 | 10.81 |
| 2-3 | 0.09 |  | -0.31 | 0.50 | 0.61 | * | 0.06 | 1.17 | -0.92 | * | -1.72 | -0.11 | -0.09 |  | -0.23 | 0.06 | -0.16 |  | -1.73 | 1.41 | 5.03 |  | -1.05 | 11.11 |
| 3-5 | 0.02 |  | -0.28 | 0.32 | 0.10 |  | -0.39 | 0.58 | -1.19 | ** | -1.90 | -0.48 | -0.11 |  | -0.23 | 0.01 | -0.71 |  | -2.01 | 0.60 | 5.50 | * | 0.19 | 10.80 |
| 6-10 | -0.09 |  | -0.40 | 0.21 | -0.40 |  | -0.97 | 0.16 | -1.40 | ** | -2.21 | -0.59 | -0.03 |  | -0.18 | 0.11 | -0.97 |  | -2.44 | 0.50 | 3.49 |  | -2.57 | 9.55 |
| 11 + | -0.03 |  | -0.33 | 0.27 | -0.55 | * | -1.09 | 0.00 | -1.50 | *** | -2.31 | -0.68 | -0.05 |  | -0.18 | 0.09 | 0.09 |  | -1.32 | 1.49 | -1.87 |  | -7.44 | 3.69 |
| **Model** | *Negative Binomial 2* | | | | *Negative Binomial 1* | | | | *Negative Binomial 1* | | | | *Negative Binomial 1* | | | | *Negative Binomial 1* | | | | *Negative Binomial 1* | | | |
| Alfa/ Delta | *0.37* |  | *0.32* | *0.42* | *6.57* |  | *6.25* | *6.91* | *9.25* |  | *8.86* | *9.66* | *1.45* |  | *1.34* | *1.57* | *533.87* |  | *510.63* | *558.17* | *305.07* |  | *283.99* | *327.71* |
| AIC | *33382* |  |  |  | *41775* |  |  |  | *25275* |  |  |  | *51976* |  |  |  | *24122* |  |  |  | *57538* |  |  |  |
| BIC | *33521* |  |  |  | *41913* |  |  |  | *25413* |  |  |  | *52114* |  |  |  | *24261* |  |  |  | *57677* |  |  |  |
|  |  |  |  |  |  |  |  |  |  |  |  |  |  |  |  |  |  |  |  |  |  |  |  |  |

*** = p < 0.05, ** = p < 0.01, *** = p < 0.001**

**r.c. = reference category**

Table S5: Regression analyses displaying the average marginal effects of the total cost of care in the different levels of the health care sector, for colorectal cancer patients’ 6 months prior to death.

|  | Secondary health care | | | | Primary health care | | | | Home- and community-based care | | | | Total health care costs | | | |
| --- | --- | --- | --- | --- | --- | --- | --- | --- | --- | --- | --- | --- | --- | --- | --- | --- |
|  | Margins (AME) |  | Confidence interval | | Margins (AME) |  | Confidence interval | | Margins (AME) |  | Confidence interval | | Margins (AME) |  | Confidence interval | |
| **Marital status** |  |  |  |  |  |  |  |  |  |  |  |  |  |  |  |  |
| Never married | r.c. |  | - | - | r.c. |  | - | - | r.c. |  | - | - | r.c. |  | - | - |
| Married | 41 535 | *** | 24 392 | 58 677 | 2 051 | *** | 992 | 3 109 | -137 604 | *** | -163 586 | -111 622 | -65 621 | *** | -87 026 | -44 215 |
| Previously married | 9 818 |  | -6 525 | 26 161 | 303 |  | -733 | 1 340 | -50 943 | *** | -78 274 | -23 612 | -20 321 |  | -42 430 | 1 788 |
| **Age** |  |  |  |  |  |  |  |  |  |  |  |  |  |  |  |  |
| Below 60 | r.c. |  | - | - | r.c. |  | - | - | r.c. |  | - | - | r.c. |  | - | - |
| 60-69 | -39 917 | *** | -59 322 | -20 511 | 326 |  | -807 | 1 460 | 18 726 | * | 4 118 | 33 335 | -31 315 | ** | -54 470 | -8 161 |
| 70-79 | -89 030 | *** | -107 768 | -70 292 | 1 067 |  | -10 | 2 144 | 50 600 | *** | 36 221 | 64 980 | -56 069 | *** | -78 660 | -33 477 |
| 80-89 | -169 269 | *** | -187 956 | -150 583 | 1 746 | ** | 669 | 2 823 | 122 489 | *** | 107 040 | 137 937 | -67 371 | *** | -90 434 | -44 307 |
| Above 90 | -223 946 | *** | -244 015 | -203 877 | -472 |  | -1 683 | 739 | 192 403 | *** | 171 811 | 212 995 | -30 342 | * | -57 555 | -3 129 |
| **Gender** |  |  |  |  |  |  |  |  |  |  |  |  |  |  |  |  |
| Men | r.c. |  | - | - | r.c. |  | - | - | r.c. |  | - | - | r.c. |  | - | - |
| Women | -4 707 |  | -13 095 | 3 681 | -931 | ** | -1 514 | -348 | 22 539 | *** | 11 506 | 33 572 | 20 171 | *** | 9 077 | 31 264 |
| **Education** |  |  |  |  |  |  |  |  |  |  |  |  |  |  |  |  |
| Primary school | r.c. |  | - | - | r.c. |  | - | - | r.c. |  | - | - | r.c. |  | - | - |
| Highschool (14 years) | 8 098 |  | -1 407 | 17 603 | -756 | * | -1 364 | -148 | -847 |  | -12 661 | 10 967 | 1 711 |  | -10 304 | 13 727 |
| Higher education | 8 400 |  | -6 192 | 22 992 | -495 |  | -1 509 | 518 | -6 940 |  | -27 112 | 13 232 | -412 |  | -18 485 | 17 661 |
| **Income** |  |  |  |  |  |  |  |  |  |  |  |  |  |  |  |  |
| 0% - 24% | r.c. |  | - | - | r.c. |  | - | - | r.c. |  | - | - | r.c. |  | - | - |
| 25% - 49% | 16 915 | ** | 5 439 | 28 392 | 801 |  | -25 | 1 627 | 2 709 |  | -14 146 | 19 563 | 21 576 | * | 4 959 | 38 193 |
| 50 % -74% | 15 858 | * | 3 733 | 27 984 | 1 068 | * | 239 | 1 897 | -15 190 |  | -31 192 | 812 | 5 615 |  | -10 371 | 21 601 |
| 75% - 100% | 23 706 | *** | 10 677 | 36 735 | 822 |  | -11 | 1 655 | -52 227 | *** | -67 468 | -36 986 | -7 055 |  | -23 211 | 9 102 |
| **Comorbidity** |  |  |  |  |  |  |  |  |  |  |  |  |  |  |  |  |
| Mild/ moderate | r.c. |  | - | - | r.c. |  | - | - | r.c. |  | - | - | r.c. |  | - | - |
| Severe | 26 496 | *** | 16 842 | 36 150 | 1 226 | *** | 539 | 1 913 | -2 861 |  | -16 638 | 10 916 | 3 752 |  | -8 755 | 16 258 |
| **Years since diagnosis** |  |  |  |  |  |  |  |  |  |  |  |  |  |  |  |  |
| 0-1 | r.c. |  | - | - | r.c. |  | - | - | r.c. |  | - | - | r.c. |  | - | - |
| 1-2 | -22 826 | *** | -34 987 | -10 665 | -36 |  | -813 | 742 | 13 103 |  | -2 352 | 28 557 | 6 213 |  | -9 759 | 22 184 |
| 2-3 | -50 123 | *** | -63 366 | -36 880 | -37 |  | -1 103 | 1 030 | 44 944 | *** | 24 480 | 65 408 | 9 372 |  | -9 451 | 28 195 |
| 3-5 | -53 831 | *** | -65 286 | -42 376 | -658 |  | -1 553 | 238 | 35 282 | *** | 18 185 | 52 378 | -1 158 |  | -17 802 | 15 486 |
| 6-10 | -56 494 | *** | -73 018 | -39 970 | -1 132 | * | -2 085 | -180 | 41 992 | *** | 22 454 | 61 530 | 11 931 |  | -6 667 | 30 529 |
| 11 + | -39 712 | *** | -52 138 | -27 287 | -850 |  | -1 736 | 36 | 42 260 | *** | 24 553 | 59 966 | 12 100 |  | -5 782 | 29 981 |
| **Model** | GLM (Identity/ gamma) | | | | GLM (Identity/ gamma) | | | | GLM (Log/ gamma) | | | | GLM (Identity/ gaussian) | | | |

*** = p < 0.05, ** = p < 0.01, *** = p < 0.001**

**r.c. = reference category**

Table S6: Regression analyses, displaying the coefficients, for colorectal cancer patients’ living situation 6 months prior to death.

|  | **Home (part 1)** | | | | **Home (part 2)** | | | | **Long term care (part 1)** | | | | **Long term care (part 2)** | | | | **Short term institution** | | | | **Hospital** | | | |
| --- | --- | --- | --- | --- | --- | --- | --- | --- | --- | --- | --- | --- | --- | --- | --- | --- | --- | --- | --- | --- | --- | --- | --- | --- |
|  | Coefficient | | Confidence interval | | Coefficient | | Confidence interval | | Coefficient | | Confidence interval | | Coefficient | | Confidence interval | | Coefficient | | Confidence interval | | Coefficient | | Confidence interval | |
| **Marital status** |  |  |  |  |  |  |  |  |  |  |  |  |  |  |  |  |  |  |  |  |  |  |  |  |
| Never married | r.c. |  | - | - | r.c. |  | - | - | r.c. |  | - | - | r.c. |  | - | - | r.c. |  | - | - | r.c. |  | - | - |
| Married | 1.51 | *** | 1.25 | 1.78 | 11.27 | *** | 7.55 | 14.99 | -1.44 | *** | -1.66 | -1.21 | -21.44 | *** | -33.51 | -9.38 | -0.58 | *** | -0.75 | -0.41 | 0.14 | *** | 0.06 | 0.22 |
| Previously married | 0.27 | * | 0.03 | 0.50 | 2.70 |  | -1.28 | 6.68 | -0.36 | ** | -0.58 | -0.15 | -1.41 |  | -11.57 | 8.75 | -0.17 | * | -0.34 | 0.00 | 0.06 |  | -0.02 | 0.14 |
| **Age** |  |  |  |  |  |  |  |  |  |  |  |  |  |  |  |  |  |  |  |  |  |  |  |  |
| Below 60 | r.c. |  | - | - | r.c. |  | - | - | r.c. |  | - | - | r.c. |  | - | - | r.c. |  | - | - | r.c. |  | - | - |
| 60-69 | -0.11 |  | -0.64 | 0.43 | 1.09 |  | -1.87 | 4.04 | 0.42 |  | -0.04 | 0.89 | -25.86 |  | -55.66 | 3.95 | 0.48 | *** | 0.23 | 0.73 | -0.14 | *** | -0.21 | -0.07 |
| 70-79 | -0.62 | * | -1.12 | -0.12 | -3.02 |  | -6.07 | 0.04 | 0.98 | *** | 0.54 | 1.42 | -14.23 |  | -41.79 | 13.34 | 0.94 | *** | 0.70 | 1.17 | -0.25 | *** | -0.33 | -0.18 |
| 80-89 | -1.30 | *** | -1.79 | -0.81 | -6.03 | *** | -9.33 | -2.73 | 1.84 | *** | 1.40 | 2.28 | -13.39 |  | -40.20 | 13.43 | 1.17 | *** | 0.94 | 1.40 | -0.60 | *** | -0.68 | -0.52 |
| Above 90 | -2.10 | *** | -2.61 | -1.59 | -11.34 | *** | -16.69 | -6.00 | 2.65 | *** | 2.19 | 3.11 | -0.58 |  | -27.84 | 26.67 | 1.06 | *** | 0.80 | 1.31 | -1.11 | *** | -1.23 | -1.00 |
| **Gender** |  |  |  |  |  |  |  |  |  |  |  |  |  |  |  |  |  |  |  |  |  |  |  |  |
| Men | r.c. |  | - | - | r.c. |  | - | - | r.c. |  | - | - | r.c. |  | - | - | r.c. |  | - | - | r.c. |  | - | - |
| Women | -0.10 |  | -0.26 | 0.05 | -4.85 | *** | -6.81 | -2.89 | 0.15 | * | 0.02 | 0.29 | -2.95 |  | -10.07 | 4.17 | 0.13 | ** | 0.04 | 0.22 | 0.00 |  | -0.04 | 0.05 |
| **Education** |  |  |  |  |  |  |  |  |  |  |  |  |  |  |  |  |  |  |  |  |  |  |  |  |
| Primary school | r.c. |  | - | - | r.c. |  | - | - | r.c. |  | - | - | r.c. |  | - | - | r.c. |  | - | - | r.c. |  | - | - |
| High school (14 years) | 0.12 |  | -0.04 | 0.28 | 0.93 |  | -1.21 | 3.08 | -0.14 | * | -0.28 | 0.00 | -1.65 |  | -8.58 | 5.27 | 0.04 |  | -0.06 | 0.13 | 0.05 | * | 0.01 | 0.10 |
| Higher education | 0.17 |  | -0.13 | 0.46 | 3.53 | * | 0.53 | 6.53 | -0.30 | * | -0.55 | -0.04 | -7.07 |  | -21.36 | 7.22 | -0.02 |  | -0.19 | 0.14 | 0.03 |  | -0.04 | 0.10 |
| **Income** |  |  |  |  |  |  |  |  |  |  |  |  |  |  |  |  |  |  |  |  |  |  |  |  |
| 0% - 24% | r.c. |  | - | - | r.c. |  | - | - | r.c. |  | - | - | r.c. |  | - | - | r.c. |  | - | - | r.c. |  | - | - |
| 25% - 49% | 0.12 |  | -0.08 | 0.32 | 1.64 |  | -1.53 | 4.81 | -0.15 |  | -0.33 | 0.03 | -3.71 |  | -12.22 | 4.80 | -0.04 |  | -0.17 | 0.09 | 0.05 |  | -0.01 | 0.12 |
| 50 % -74% | 0.18 |  | -0.03 | 0.40 | 2.42 |  | -0.57 | 5.41 | -0.35 | *** | -0.54 | -0.17 | -1.57 |  | -10.74 | 7.61 | 0.00 |  | -0.13 | 0.14 | 0.06 |  | 0.00 | 0.13 |
| 75% - 100% | 0.67 | *** | 0.43 | 0.91 | 2.14 |  | -0.82 | 5.09 | -0.68 | *** | -0.89 | -0.47 | -9.90 |  | -20.83 | 1.04 | -0.15 | * | -0.29 | -0.01 | 0.11 | ** | 0.05 | 0.18 |
| **Comorbidity** |  |  |  |  |  |  |  |  |  |  |  |  |  |  |  |  |  |  |  |  |  |  |  |  |
| Mild/ moderate | r.c. |  | - | - | r.c. |  | - | - | r.c. |  | - | - | r.c. |  | - | - | r.c. |  | - | - | r.c. |  | - | - |
| Severe | 0.86 | *** | 0.64 | 1.07 | 1.54 |  | -0.59 | 3.68 | -0.53 | *** | -0.70 | -0.36 | -30.73 | *** | -40.68 | -20.79 | 0.20 | *** | 0.09 | 0.31 | 0.04 |  | -0.01 | 0.09 |
| **Years since diagnosis** |  |  |  |  |  |  |  |  |  |  |  |  |  |  |  |  |  |  |  |  |  |  |  |  |
| 0-1 | r.c. |  | - | - | r.c. |  | - | - | r.c. |  | - | - | r.c. |  | - | - | r.c. |  | - | - | r.c. |  | - | - |
| 1-2 | -0.27 | * | -0.50 | -0.04 | -2.02 |  | -4.76 | 0.71 | 0.31 | ** | 0.11 | 0.51 | 4.24 |  | -6.33 | 14.82 | 0.02 |  | -0.12 | 0.16 | -0.17 | *** | -0.23 | -0.11 |
| 2-3 | -0.62 | *** | -0.89 | -0.36 | -1.34 |  | -4.59 | 1.90 | 0.48 | *** | 0.25 | 0.72 | 8.25 |  | -4.01 | 20.52 | 0.14 |  | -0.02 | 0.30 | -0.29 | *** | -0.36 | -0.21 |
| 3-5 | -0.56 | *** | -0.79 | -0.34 | -1.03 |  | -4.01 | 1.94 | 0.47 | *** | 0.27 | 0.66 | 11.91 | * | 1.93 | 21.88 | 0.03 |  | -0.12 | 0.17 | -0.26 | *** | -0.32 | -0.20 |
| 6-10 | -0.70 | *** | -0.95 | -0.46 | -3.32 |  | -6.79 | 0.16 | 0.58 | *** | 0.36 | 0.80 | 17.47 | ** | 6.82 | 28.12 | 0.07 |  | -0.09 | 0.24 | -0.25 | *** | -0.32 | -0.18 |
| 11 + | -0.59 | *** | -0.81 | -0.37 | -3.07 |  | -6.65 | 0.51 | 0.51 | *** | 0.31 | 0.71 | 14.38 | ** | 5.06 | 23.69 | -0.01 |  | -0.16 | 0.14 | -0.20 | *** | -0.27 | -0.12 |
| **Model** | *Logistic regression* | | | | *OLS* | | | | *Logistic regression* | | | | *OLS* | | | | *Negative Binomial 1* | | | | *Negative Binomial 1* | | | |
| Alfa/ Delta** |  |  |  |  |  |  |  |  |  |  |  |  |  |  |  |  | 6.53 |  | 6.26 | 6.81 | *18.44* |  | *17.69* | *19.21* |
| AIC | *69533* | | | | | | | | *24160* | | | | | | | | *43670* |  |  |  | *59585* |  |  |  |
| BIC | *69796* | | | | | | | | *24423* | | | | | | | | *43808* |  |  |  | *59723* |  |  |  |

*** = p < 0.05, ** = p < 0.01, *** = p < 0.001**

Table S7: Regression analyses, displaying the coefficients, for colorectal cancer patients’ health care utilization 6 months prior to death.

|  | Inpatient treatment | | | | Outpatient treatment | | | | GP consultations | | | | ER visits | | | | Practical assistance (hours) | | | | Home nursing (hours) | | | |
| --- | --- | --- | --- | --- | --- | --- | --- | --- | --- | --- | --- | --- | --- | --- | --- | --- | --- | --- | --- | --- | --- | --- | --- | --- |
|  | Coefficient | | Confidence interval | | Coefficient | | Confidence interval | | Coefficient | | Confidence interval | | Coefficient | | Confidence interval | | Coefficient | | Confidence interval | | Coefficient | | Confidence interval | |
| **Marital status** |  |  |  |  |  |  |  |  |  |  |  |  |  |  |  |  |  |  |  |  |  |  |  |  |
| Never married | r.c. |  | - | - | r.c. |  | - | - | r.c. |  | - | - | r.c. |  | - | - | r.c. |  | - | - | r.c. |  | - | - |
| Married | 0.08 |  | -0.05 | 0.20 | 0.37 | *** | 0.28 | 0.46 | 0.22 | *** | 0.14 | 0.29 | -0.04 |  | -0.14 | 0.06 | -1.24 | *** | -1.42 | -1.06 | -0.12 | * | -0.24 | -0.01 |
| Previously married | -0.01 |  | -0.12 | 0.11 | 0.14 | ** | 0.05 | 0.23 | 0.08 | * | 0.00 | 0.16 | 0.06 |  | -0.04 | 0.16 | -0.11 |  | -0.27 | 0.05 | -0.01 |  | -0.13 | 0.11 |
| **Age** |  |  |  |  |  |  |  |  |  |  |  |  |  |  |  |  |  |  |  |  |  |  |  |  |
| Below 60 | r.c. |  | - | - | r.c. |  | - | - | r.c. |  | - | - | r.c. |  | - | - | r.c. |  | - | - | r.c. |  | - | - |
| 60-69 | -0.07 |  | -0.16 | 0.02 | -0.19 | *** | -0.26 | -0.12 | 0.01 |  | -0.07 | 0.08 | 0.01 |  | -0.11 | 0.13 | -0.05 |  | -0.31 | 0.21 | 0.08 |  | -0.05 | 0.21 |
| 70-79 | -0.23 | *** | -0.32 | -0.14 | -0.43 | *** | -0.51 | -0.36 | 0.08 | * | 0.01 | 0.16 | 0.15 | * | 0.03 | 0.26 | 0.32 | * | 0.07 | 0.57 | 0.15 | * | 0.02 | 0.28 |
| 80-89 | -0.53 | *** | -0.62 | -0.43 | -1.01 | *** | -1.09 | -0.93 | 0.07 |  | -0.01 | 0.14 | 0.20 | ** | 0.09 | 0.32 | 0.72 | *** | 0.46 | 0.97 | 0.44 | *** | 0.30 | 0.57 |
| Above 90 | -0.94 | *** | -1.06 | -0.83 | -1.51 | *** | -1.62 | -1.39 | -0.21 | *** | -0.32 | -0.11 | 0.12 |  | -0.02 | 0.26 | 0.82 | *** | 0.54 | 1.10 | 0.37 | *** | 0.20 | 0.53 |
| **Gender** |  |  |  |  |  |  |  |  |  |  |  |  |  |  |  |  |  |  |  |  |  |  |  |  |
| Men | r.c. |  | - | - | r.c. |  | - | - | r.c. |  | - | - | r.c. |  | - | - | r.c. |  | - | - | r.c. |  | - | - |
| Women | -0.02 |  | -0.07 | 0.03 | -0.04 |  | -0.08 | 0.00 | -0.01 |  | -0.05 | 0.03 | -0.07 | * | -0.12 | -0.01 | 0.58 | *** | 0.47 | 0.69 | 0.14 | *** | 0.07 | 0.21 |
| **Education** |  |  |  |  |  |  |  |  |  |  |  |  |  |  |  |  |  |  |  |  |  |  |  |  |
| Primary school | r.c. |  | - | - | r.c. |  | - | - | r.c. |  | - | - | r.c. |  | - | - | r.c. |  | - | - | r.c. |  | - | - |
| High school (14 years) | 0.04 |  | -0.02 | 0.10 | 0.11 | *** | 0.06 | 0.15 | -0.05 | * | -0.10 | -0.01 | -0.13 | *** | -0.19 | -0.07 | 0.09 |  | -0.02 | 0.20 | -0.01 |  | -0.08 | 0.06 |
| Higher education | 0.05 |  | -0.04 | 0.14 | 0.19 | *** | 0.12 | 0.26 | -0.09 | ** | -0.16 | -0.03 | -0.20 | *** | -0.30 | -0.11 | 0.17 |  | -0.01 | 0.36 | -0.01 |  | -0.12 | 0.10 |
| **Income** |  |  |  |  |  |  |  |  |  |  |  |  |  |  |  |  |  |  |  |  |  |  |  |  |
| 0% - 24% | r.c. |  | - | - | r.c. |  | - | - | r.c. |  | - | - | r.c. |  | - | - | r.c. |  | - | - | r.c. |  | - | - |
| 25% - 49% | -0.01 |  | -0.11 | 0.08 | 0.05 |  | -0.02 | 0.12 | 0.08 | ** | 0.02 | 0.14 | -0.06 |  | -0.14 | 0.01 | -0.04 |  | -0.19 | 0.10 | 0.07 |  | -0.03 | 0.17 |
| 50 % -74% | -0.01 |  | -0.09 | 0.08 | 0.16 | *** | 0.09 | 0.22 | 0.11 | *** | 0.05 | 0.17 | -0.06 |  | -0.13 | 0.02 | -0.01 |  | -0.16 | 0.13 | 0.07 |  | -0.02 | 0.17 |
| 75% - 100% | 0.02 |  | -0.07 | 0.11 | 0.25 | *** | 0.18 | 0.32 | 0.08 | * | 0.02 | 0.14 | -0.17 | *** | -0.25 | -0.08 | -0.24 | ** | -0.40 | -0.08 | 0.00 |  | -0.10 | 0.10 |
| **Comorbidity** |  |  |  |  |  |  |  |  |  |  |  |  |  |  |  |  |  |  |  |  |  |  |  |  |
| Mild/ moderate | r.c. |  | - | - | r.c. |  | - | - | r.c. |  | - | - | r.c. |  | - | - | r.c. |  | - | - | r.c. |  | - | - |
| Severe | 0.15 | *** | 0.08 | 0.22 | 0.49 | *** | 0.44 | 0.54 | 0.17 | *** | 0.12 | 0.21 | -0.01 |  | -0.08 | 0.05 | 0.22 | *** | 0.10 | 0.34 | 0.49 | *** | 0.41 | 0.57 |
| **Years since diagnosis** |  |  |  |  |  |  |  |  |  |  |  |  |  |  |  |  |  |  |  |  |  |  |  |  |
| 0-1 | r.c. |  | - | - | r.c. |  | - | - | r.c. |  | - | - | r.c. |  | - | - | r.c. |  | - | - | r.c. |  | - | - |
| 1-2 | -0.02 |  | -0.08 | 0.04 | 0.14 | *** | 0.08 | 0.20 | -0.03 |  | -0.08 | 0.03 | 0.01 |  | -0.07 | 0.08 | -0.04 |  | -0.19 | 0.10 | 0.11 | * | 0.01 | 0.20 |
| 2-3 | 0.03 |  | -0.09 | 0.14 | 0.08 | * | 0.01 | 0.15 | -0.08 | * | -0.14 | -0.01 | -0.06 |  | -0.15 | 0.04 | -0.02 |  | -0.19 | 0.16 | 0.10 |  | -0.02 | 0.21 |
| 3-5 | 0.01 |  | -0.08 | 0.09 | 0.01 |  | -0.05 | 0.08 | -0.10 | ** | -0.16 | -0.04 | -0.07 |  | -0.15 | 0.01 | -0.08 |  | -0.23 | 0.07 | 0.10 | * | 0.00 | 0.20 |
| 6-10 | -0.03 |  | -0.12 | 0.06 | -0.06 |  | -0.14 | 0.02 | -0.12 | ** | -0.19 | -0.05 | -0.02 |  | -0.11 | 0.07 | -0.11 |  | -0.29 | 0.06 | 0.07 |  | -0.05 | 0.18 |
| 11 + | -0.01 |  | -0.10 | 0.08 | -0.08 |  | -0.16 | 0.00 | -0.13 | *** | -0.20 | -0.06 | -0.03 |  | -0.12 | 0.06 | 0.01 |  | -0.15 | 0.16 | -0.04 |  | -0.15 | 0.08 |
| **Model** | *Negative Binomial 2* | | | | *Negative Binomial 1* | | | | *Negative Binomial 1* | | | | *Negative Binomial 1* | | | | *Negative Binomial 1* | | | | *Negative Binomial 1* | | | |
| Alfa/ Delta | *0.37* |  | *0.32* | *0.42* | *6.57* |  | *6.25* | *6.91* | *9.25* |  | *8.86* | *9.66* | *1.45* |  | *1.34* | *1.57* | *133.78* |  | *113.29* | *157.99* | *305.07* |  | *283.99* | *327.71* |
| AIC | *33382* |  |  |  | *41775* |  |  |  | *25275* |  |  |  | *51976* |  |  |  | *24122* |  |  |  | *57538* |  |  |  |
| BIC | *33521* |  |  |  | *41913* |  |  |  | *25413* |  |  |  | *52114* |  |  |  | *24261* |  |  |  | *57677* |  |  |  |

*** = p < 0.05, ** = p < 0.01, *** = p < 0.001**

**r.c. = reference category**

Table S8: Results when running our models with different specifications of the Charlson Comorbidity Index. Numbers presented as average marginal effects.

|  | **Method 1** | **Method 2** | **Method 3** |
| --- | --- | --- | --- |
| **Home (days)** |  |  |  |
| *Married* | 30,42 | 30,15 | 30,49 |
| *Previously married* | 7,06 | 6,83 | 7,10 |
| **Long-term institution (days)** |  |  |  |
| *Married* | -29,16 | -28,71 | -29,23 |
| *Previously married* | -8,42 | -8,07 | -8,46 |
| **Short-term institution (days)** |  |  |  |
| *Married* | -10,12 | -10,52 | -10,23 |
| *Previously married* | -3,65 | -3,88 | -3,74 |
| **Hospital (days)** |  |  |  |
| *Married* | 2,90 | 2,78 | 2,87 |
| *Previously married* | 1,21 | 1,17 | 1,18 |
| **Inpatient stay** |  |  |  |
| *Married* | 0,26 | 0,24 | 0,24 |
| *Previously married* | -0,02 | -0,03 | -0,03 |
| **Outpatient treatments** |  |  |  |
| *Married* | 2,57 | 2,50 | 2,55 |
| *Previously married* | 0,89 | 0,83 | 0,88 |
| **GP consultations** |  |  |  |
| *Married* | 2,49 | 2,38 | 2,46 |
| *Previously married* | 0,84 | 0,81 | 0,81 |
| **ER-visits** |  |  |  |
| *Married* | -0,06 | -0,07 | -0,07 |
| *Previously married* | 0,09 | 0,09 | 0,09 |
| **Home nursing (hours)** |  |  |  |
| *Married* | -6,59 | -7,62 | -6,82 |
| *Previously married* | -0,52 | -1,07 | -0,70 |
| **Practical assistance (hours)** |  |  |  |
| *Married* | -9,36 | -9,51 | -9,43 |
| *Previously married* | -1,39 | -1,49 | -1,46 |
| **Costs secondary care** |  |  |  |
| *Married* | 41 535 | 43 147 | 41 945 |
| *Previously married* | 9 818 | 11 531 | 10 233 |
| **Costs primary care** |  |  |  |
| *Married* | 2 051 | 2 002 | 2 026 |
| *Previously married* | 303 | 273 | 292 |
| **Costs home and community-based care** |  |  |  |
| *Married* | -137 604 | -139 203 | -138 353 |
| *Previously married* | -50 943 | -51 712 | -51 694 |
| **Total costs** |  |  |  |
| *Married* | -65 621 | -66 602 | -66 226 |
| *Previously married* | -20 321 | -20 615 | -20 757 |

**Method 1 = Using the Charlso Index as in the original paper (mild/ moderate or severe)**

**Method 1 = Using the Charlso Index as a continuous scale**

**Method 1 = Using three groups of the Charlso Index: mild (values 0 – 2), moderate (3 – 4) and severe (> 5)**
